# Supplementary material for: Agronomic Linked Data (AgroLD): A knowledge-based system to enable integrative biology in agronomy
Source: PLoS One. 2018 Nov 30;13(11):e0198270. doi: 10.1371/journal.pone.0198270 (PMC6269127; doi:10.1371/journal.pone.0198270)
Supplement: S1 File — This document shows how to use the various features of the platform. (PDF) [file pone.0198270.s001.pdf]

AgroLD User Guide

agrold.org

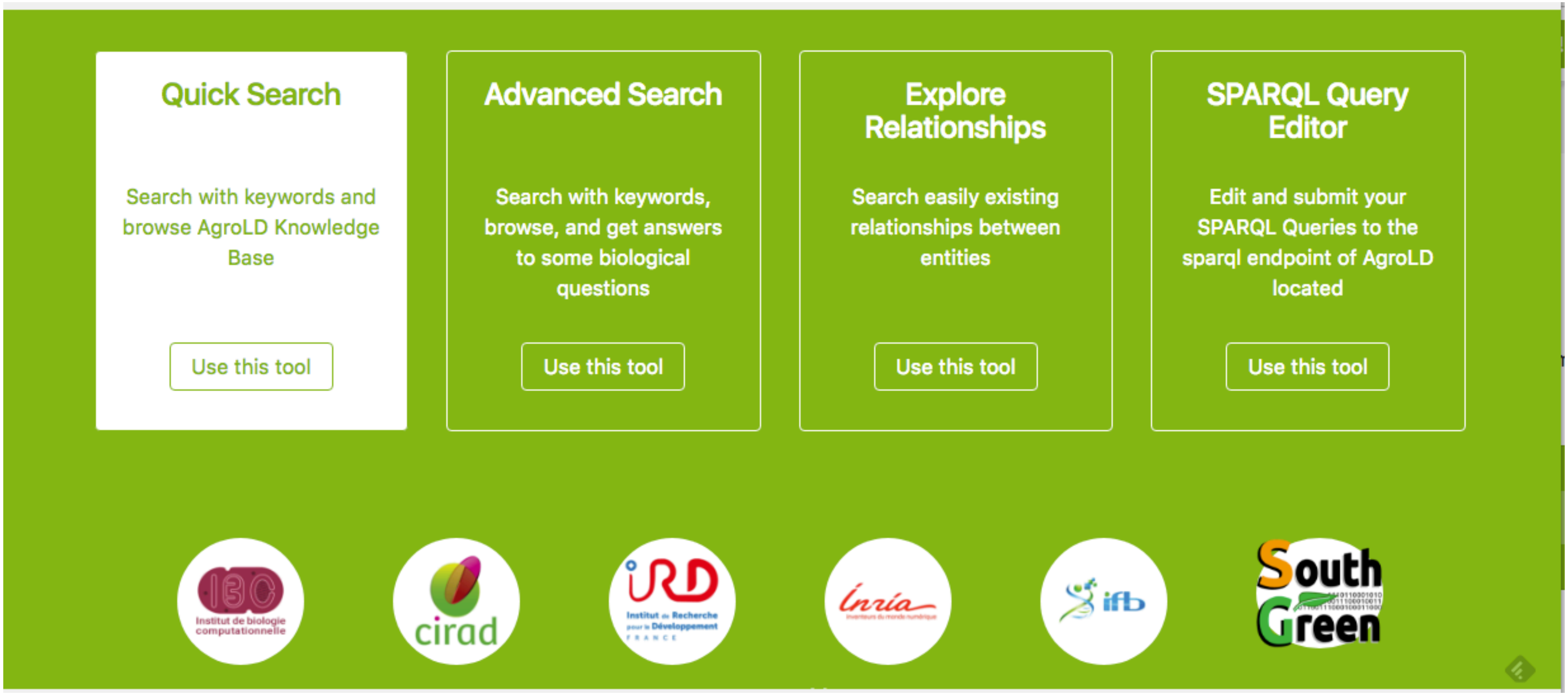

Quick Search
Search with keywords and browse AgroLD Knowledge Base
Use this tool
Advanced Search
Search with keywords, browse, and get answers to some biological questions
Use this tool
Explore Relationships
Search easily existing relationships between entities
Use this tool
SPARQL Query Editor
Edit and submit your SPARQL Queries to the sparql endpoint of AgroLD located
Use this tool
Institut de biologie computationnelle
cirad
Institut de Recherche pour le Développement FRANCE
Inria
ifb
South Green

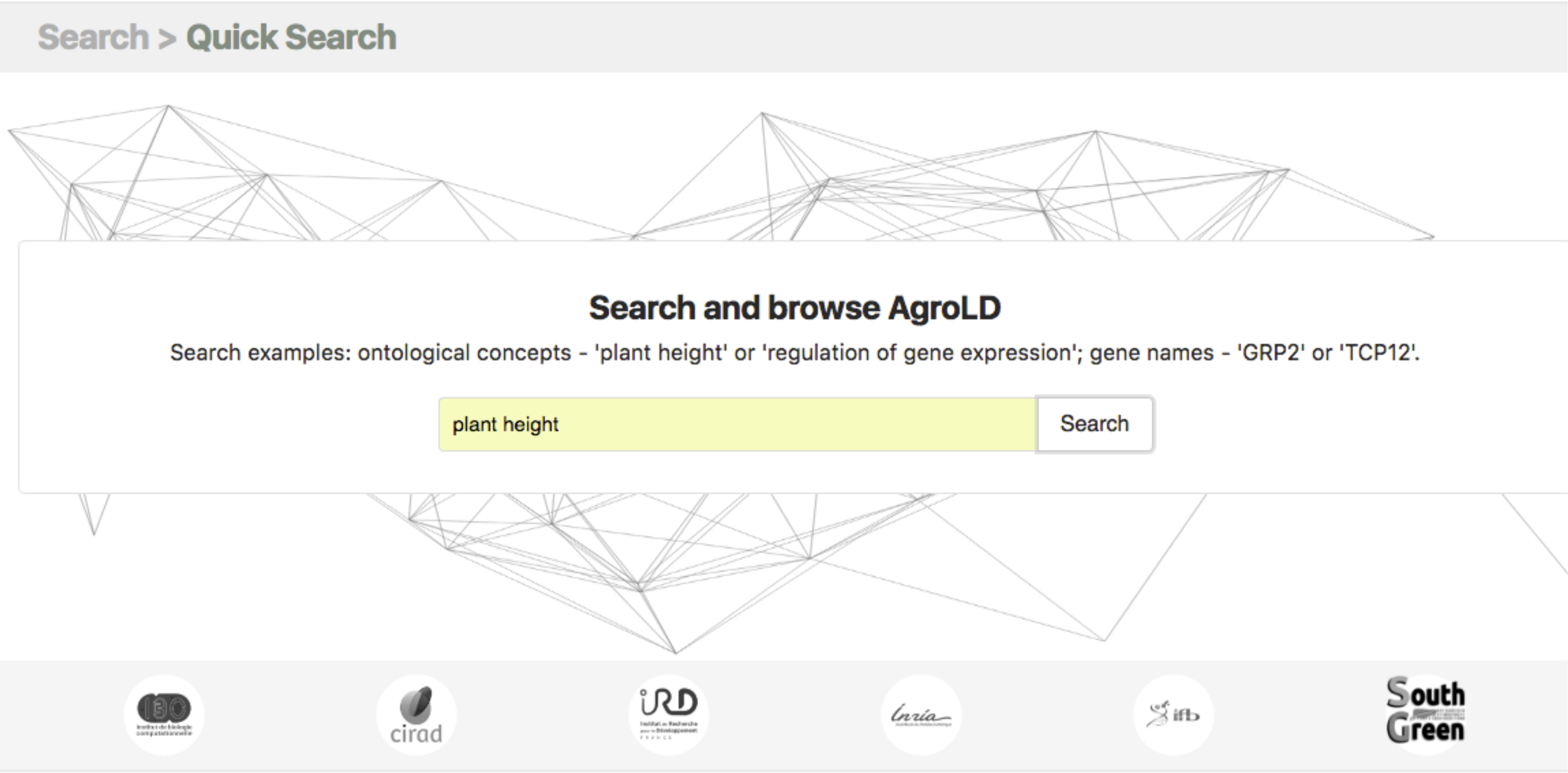
Search > Quick Search
Search and browse AgroLD
Search examples: ontological concepts - 'plant height' or 'regulation of gene expression'; gene names - 'GRP2' or 'TCP12'.
plant height
Search
cirad
South
Green

How to refine Results

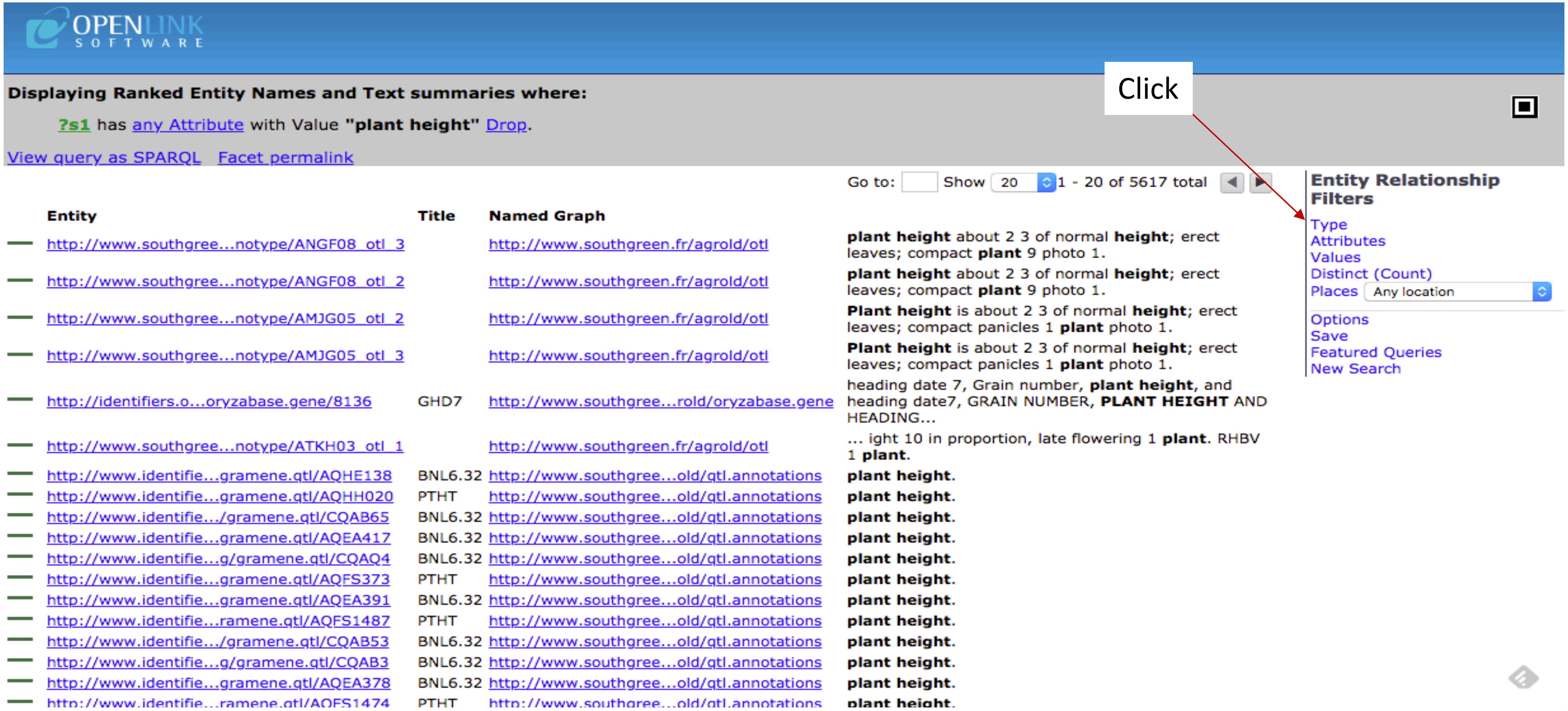

Select the types you want to display

Select only Gene type for instance

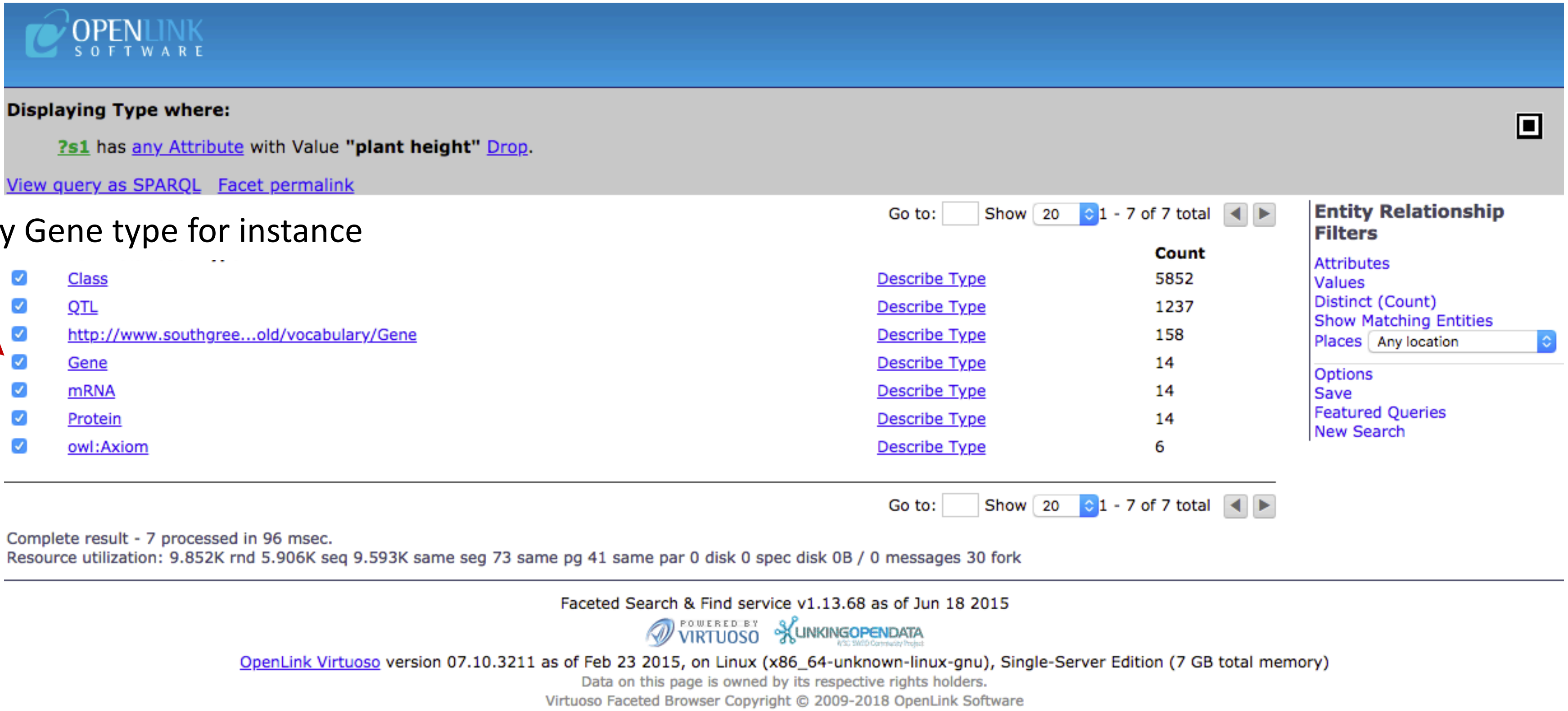

Results are displayed

Displaying Ranked Entity Names and Text summaries where:

?s1 has any Attribute with Value **"plant height"** Drop.
?s1 is not a **owl:Class** . Drop
?s1 is not a **http://www.southgree...agrold/resource/QTL** . Drop
?s1 is not a **http://www.southgree...grold/resource/mRNA** . Drop
?s1 is not a **owl:Axiom** . Drop

Link to the gene

RQL Facet permali

Name of the gene in that case

Source of the data

Matching key word in the contextual text

Go to: Show 20 1 - 20 of 73 total

Entity	Title	Named Graph	
http://identifiers.o...oryzabase.gene/8136	GHD7	http://www.southgree...rold/oryzabase.gene	heading date 7, Grain number, **plant height**, and heading date7, GRAIN NUMBER, **PLANT HEIGHT** AND HEADING...
http://identifiers.o....plant/Os03g0786400	Os03g0786400	http://www.southgree...r/agrold/qtaro.gene	**Plant height**.
http://identifiers.o....plant/Os01g0626400	Os01g0626400	http://www.southgree...r/agrold/qtaro.gene	**plant height** through control of cell size.
http://identifiers.o....plant/Os01g0940000	Os01g0940000	http://www.southgree...r/agrold/qtaro.gene	**plant height**.
http://identifiers.o...oryzabase.gene/5063	qPH-7-1(t) (qPHT1-1)	http://www.southgree...rold/oryzabase.gene	**plant height** QTL 7 1 t **plant height** QTL 7 1 t.
http://identifiers.o....plant/Os05g0591400	Os05g0591400	http://www.southgree...r/agrold/qtaro.gene	**Plant height**.
http://identifiers.o...oryzabase.gene/5062	qPH-3-6(t) (qPHT3-6)	http://www.southgree...rold/oryzabase.gene	**plant height** QTL 3 6 t **plant height** QTL 3 6 t.
http://identifiers.o...oryzabase.gene/5061	qPH-3-5(t) (qPHT3-5)	http://www.southgree...rold/oryzabase.gene	**plant height** QTL 3 5 t **plant height** QTL 3 5 t.
http://identifiers.o...oryzabase.gene/6613	PH1	http://www.southgree...rold/oryzabase.gene	**PLANT HEIGHT** 1Plant **height**...
http://identifiers.o.../oryzabase.gene/424	D26	http://www.southgree...rold/oryzabase.gene	**Plant height** is about two thirds of normal type.
http://identifiers.o...oryzabase.gene/6612	PH2	http://www.southgree...rold/oryzabase.gene	**PLANT HEIGHT** 2Plant **height**...
http://identifiers.o.../oryzabase.gene/615	D31	http://www.southgree...rold/oryzabase.gene	**Plant height** is about 70 cm with narrow and dark green leaves.
http://identifiers.o...oryzabase.gene/6611	PH3	http://www.southgree...rold/oryzabase.gene	**PLANT HEIGHT** 3Plant **height**...
http://identifiers.o...oryzabase.gene/6364	qPh	http://www.southgree...rold/oryzabase.gene	**plant** HEIGHT.
http://identifiers.o...oryzabase.gene/6610	PH4	http://www.southgree...rold/oryzabase.gene	**PLANT HEIGHT** 4Plant **height**...
http://identifiers.o...oryzabase.gene/2443	qPH-5-2(t) (ph5b)	http://www.southgree...rold/oryzabase.gene	**plant height** QTL 5 2 t This QTL controls **plant height**, being located on chromosome 5 flanked by DNA markers... **plant height** QTL 5 2 t.

Following the link of the Entity (e.g. Gene)

About: GHD7 Goto Sponge NotDistinct Permalink

An Entity of Type : http://www.southgreen.fr/agrold/vocabulary/Gene, within Data Space : volvestre.cirad.fr:8890 associated with source document(s)

Type: http://www.southgreen.fr/agrold/vocabulary/Gene Command: Start New Facet Go

Direct Link to the original source

Select this Link to display all genes tagged with this trait

Attributes	Values
rdf:type	http://www.southgreen.fr/agrold/vocabulary/Gene
rdfs:label	GHD7
rdfs:subClassOf	SO:0000704
description	HEADING DATE 7
has synonym	Ghd7<br>Ghd2
has trait	inflorescence branching<br>days to heading<br>plant height<br>grain number<br>TO_0000050<br>TO_0000137<br>TO_0000207<br>TO_0002759
http://www.southgr...bulary/chromosome	7
http://www.southgr...ulary/explanation	EU286800(Minghui 63 genomic), EU286801(Minghui 63 mRNA). OsI_25572. CCT domain protein. JF926532-JF926543 (Indica and Japonica). flowering time gene.
http://www.southgr...ocabulary/go_term	response to light stimulus<br>photoperiodism<br>photoperiodism, flowering
http://www.southgr.../has_protein_name	CCT DOMAIN PROTEIN
http://www.southgr...as_rap_identifier	http://www.southgreen.fr/agrold/rapdb/Os07g0261200
http://www.southgr...y/has_trait_class	Heterochrony,Yield and productivity,Heading date
http://www.southgr...d/vocabulary/name	heading date 7, Grain number, plant height, and heading date7, GRAIN NUMBER, PLANT HEIGHT AND HEADING DATE 7
http://www.southgr..._alternative_name	CCT domain protein
http://www.southgr...bulary/has_allele	Ghd7-0<br>Ghd7-0a<br>Ghd7-1<br>Ghd7-2<br>Ghd7-3

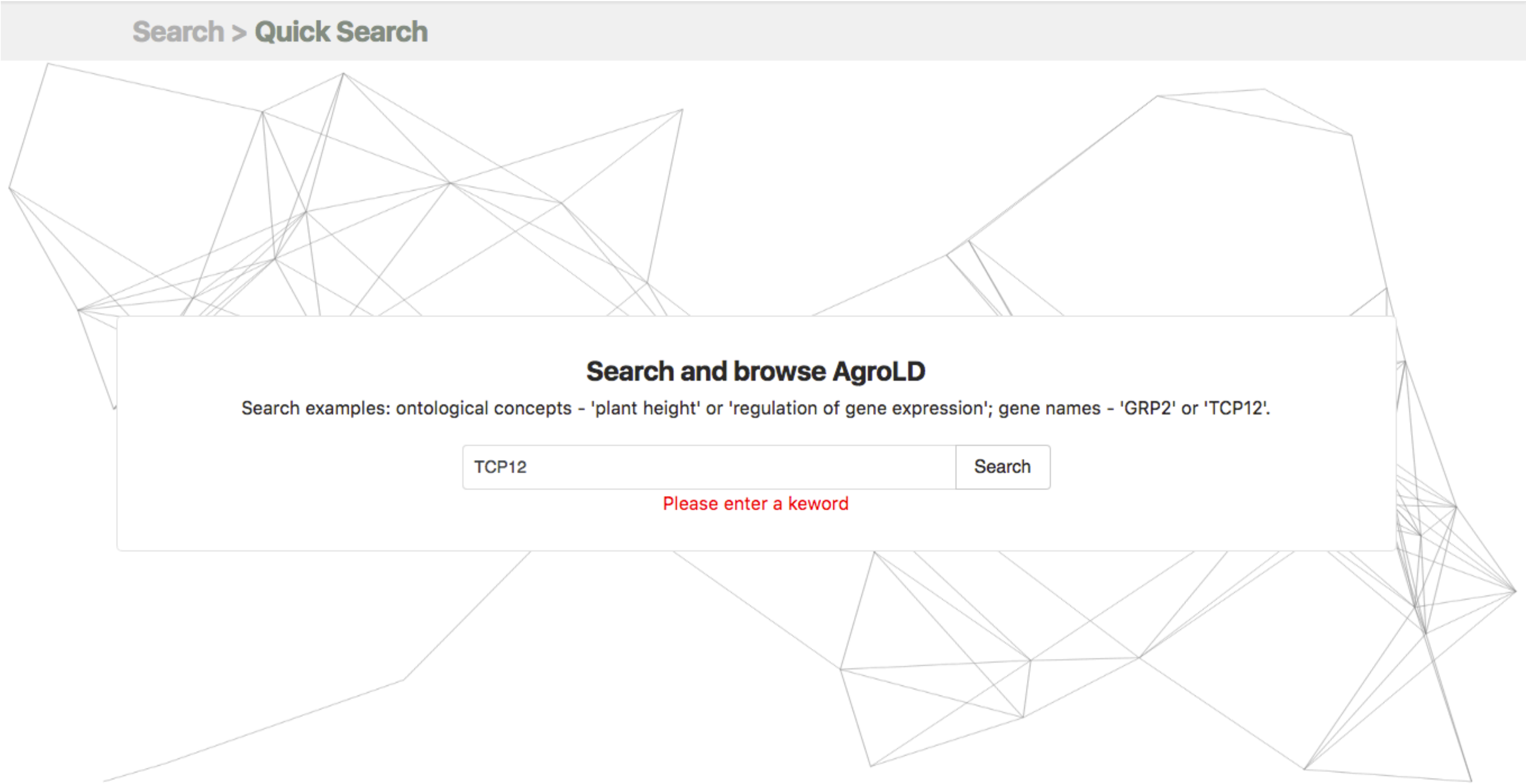
Search > Quick Search
Search and browse AgroLD
Search examples: ontological concepts - 'plant height' or 'regulation of gene expression'; gene names - 'GRP2' or 'TCP12'.
TCP12
Search
Please enter a keword

Direct Link to the display the entity

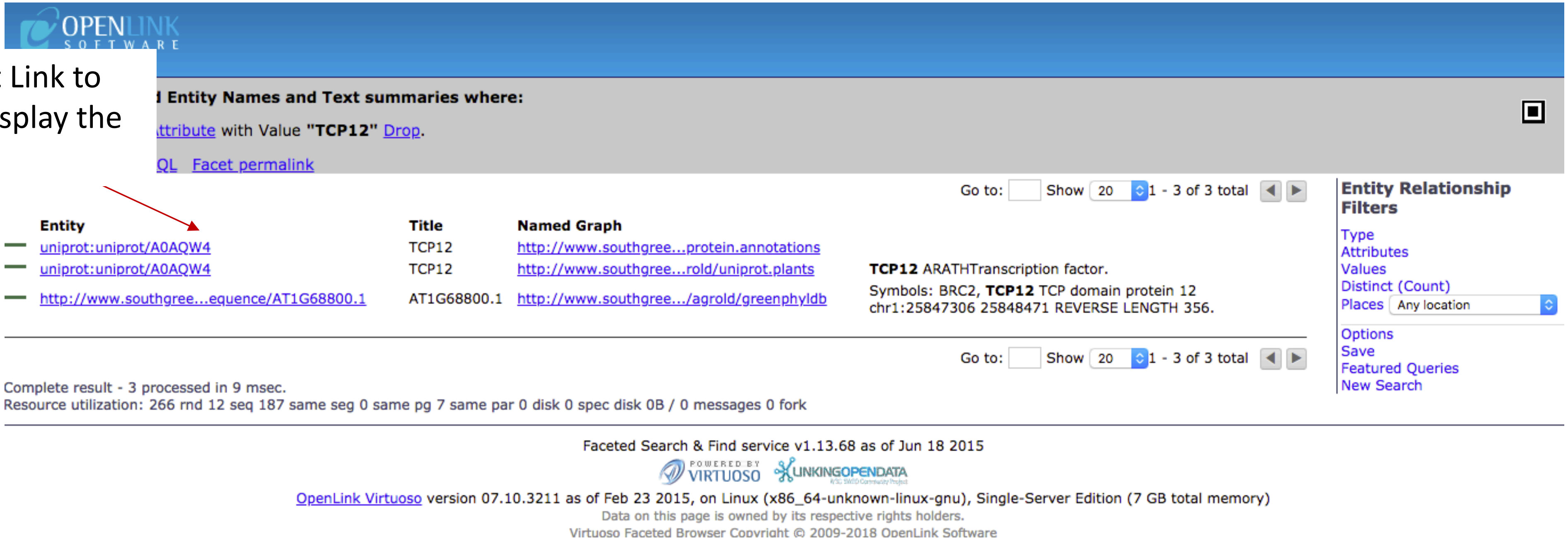

About: TCP12 Goto Sponge NotDistinct Permalink

An Entity of Type : http://www.southgreen.fr/agrold/resource/Protein, within Data Space : volvestre.cirad.fr:8890 associated with source document(s)

Command: Start New Facet Go

Attributes	Values
rdf:type	Class<br>Protein
rdfs:label	TCP12<br>TCP12_ARATH
rdfs:subClassOf	SO:0000104
description	Transcription factor TCP12
classified with	Complete proteome<br>Reference proteome<br>Developmental protein<br>Nucleus<br>DNA-binding<br>»more»
taxon	Arabidopsis thaliana
has dbxref	uniprot:interpro/IPR017888<br>uniprot:prosite/PS51370<br>uniprot:biogrid/28433<br>uniprot:eggnog/NOG266795<br>uniprot:embl/AM408561<br>uniprot:ensemblplants/AT1G68800.1<br>uniprot:expressionatlas/A0AQW4<br>uniprot:geneid/843212<br>uniprot:genevestigator/A0AQW4<br>uniprot:hogenom/HOG000154526<br>uniprot:inparanoid/A0AQW4<br>uniprot:kegg/ath:AT1G68800<br>uniprot:oma/ERTMAKM<br>uniprot:phylomedb/A0AQW4<br>uniprot:pir/G96712<br>uniprot:pride/A0AQW4<br>uniprot:proteinmodelportal/A0AQW4<br>uniprot:refseq/NP_177047.2<br>uniprot:string/3702.AT1G68800.1-P<br>uniprot:tair/AT1G68800<br>uniprot:unigene/At.64893<br>uniprot:embl/AC011914<br>uniprot:proteomes/UP000006548<br>uniprot:embl/CP002684<br>uniprot:interpro/IPR005333<br>uniprot:interpro/IPR017887<br>uniprot:pfam/PF03634<br>uniprot:prosite/PS51369
[illegible]	[illegible]

Direct Link to the original source

has annotation	http://identifiers.org/goa/A0AQW4
has function	DNA binding<br>sequence-specific DNA binding transcription factor activity
has synonym	BRC2<br>At1g68800<br>F14K14.9<br>TCP12<br>TCP12_ARATH
located in	nucleus
participates in	transcription, DNA-dependent<br>regulation of transcription, DNA-dependent<br>multicellular organismal development<br>regulation of secondary shoot formation
has symbol	TCP12
comment	FUNCTION: Transcription factor that prevents axillary bud outgrowth. May also delay early axillary bud development. Probably involved in the auxin-indu {ECO:0000269|PubMed:17307924}.SUBCELLULAR LOCATION: Nucleus {ECO:0000255|PROSITE- ProRule:PRU00701, ECO:0000269|PubMed:17307924} and, to a lower extent, in axillary structures such as flowers and siliques. {ECO:0000269|PubMed:17307924}.DEVELOPMENTAL STAGE: Detected in axill after flowering. During bud vegetative development, down-regulated in the outer layers of the meristem, but accumulates transiently in young leaf primo provascular tissue underlying the bud. Accumulates in axillary buds, but disappears at the time of bud outgrowth. {ECO:0000269|PubMed:17307924}.IN meristem (SAM) decapitation (release from apical dominance). {ECO:0000269|PubMed:17307924}.SIMILARITY: Contains 1 R domain. {ECO:0000255|P 1 TCP domain. {ECO:0000255|PROSITE- ProRule:PRU00701}.SEQUENCE CAUTION: Sequence=AAG52043.1; Type=Erroneous gene model prediction; E
has alternative id	uniprot:uniprot/Q9CA43
is encodes of	BRC2
is has dbxref of	AT1G68800.1
is rdf:subject of	http://identifiers.org/goa/A0AQW4

Link to the Gene

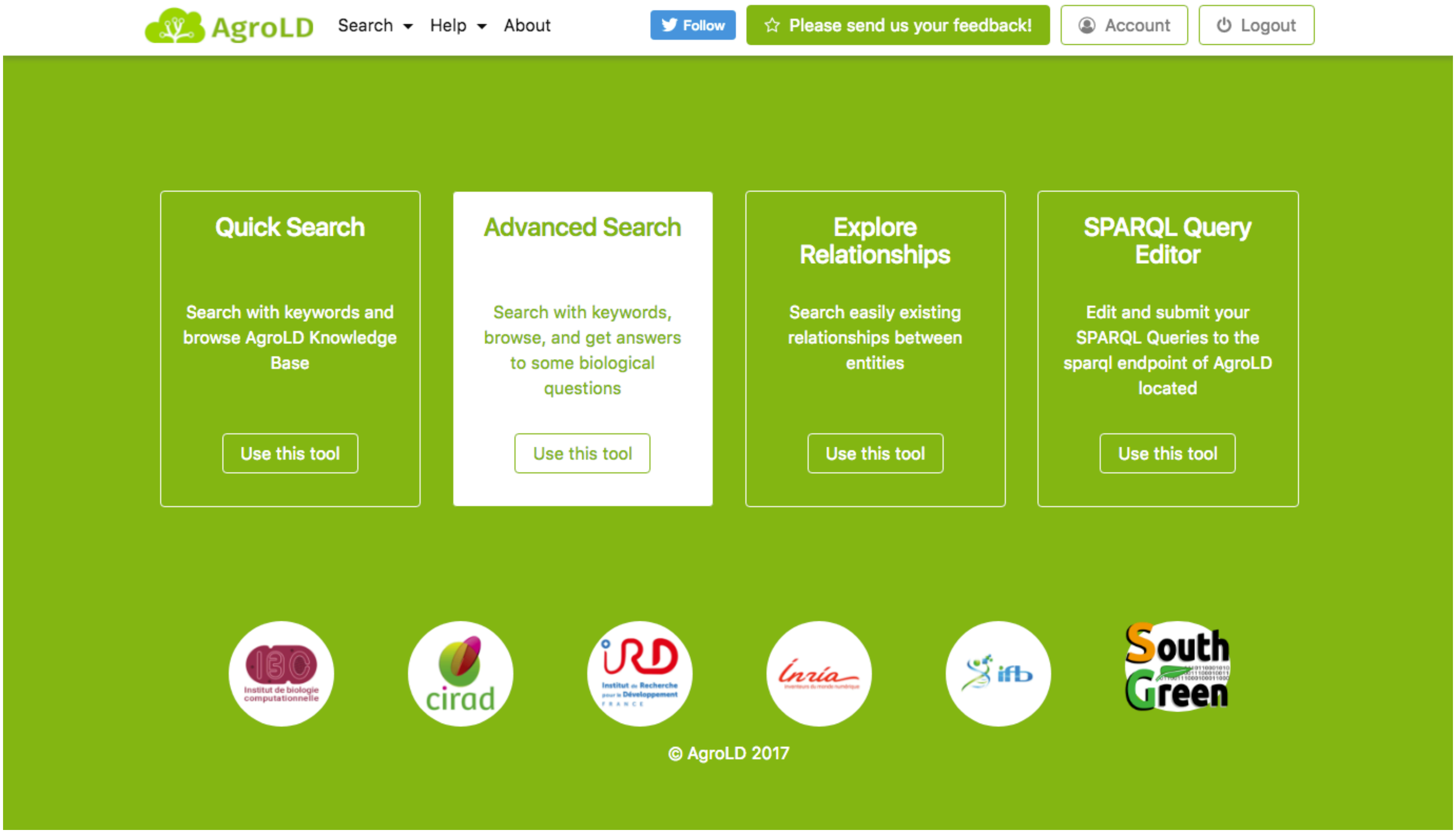
AgroLD
Search
Help
About
Follow
Please send us your feedback!
Account
Logout
Quick Search
Search with keywords and browse AgroLD Knowledge Base
Use this tool
Advanced Search
Search with keywords, browse, and get answers to some biological questions
Use this tool
Explore Relationships
Search easily existing relationships between entities
Use this tool
SPARQL Query Editor
Edit and submit your SPARQL Queries to the sparql endpoint of AgroLD located
Use this tool
Institut de biologie computationnelle
cirad
Institut de Recherche pour le Développement
South Green
© AgroLD 2017

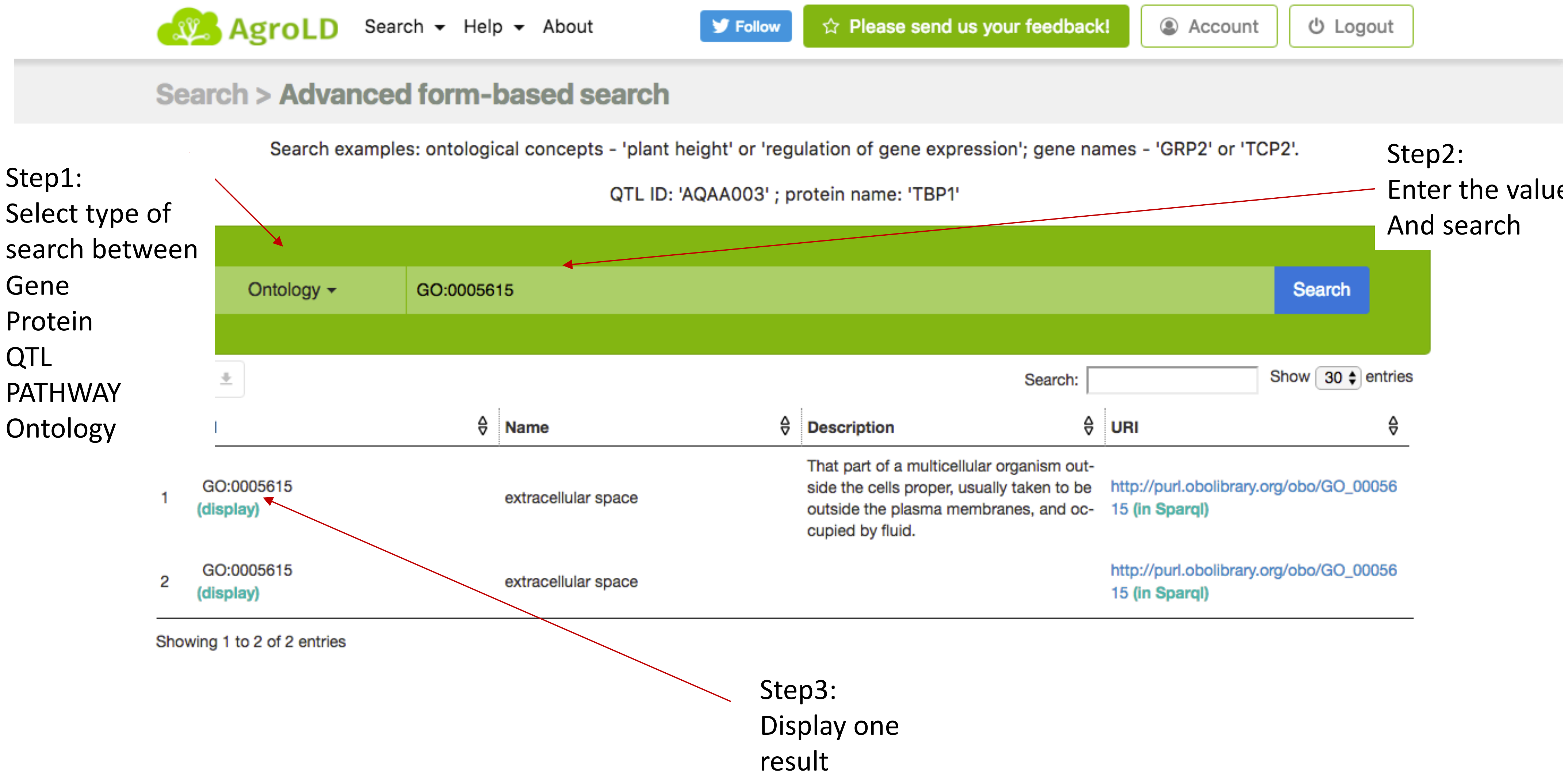
AgroLD
Search
Help
About
Follow
Please send us your feedback!
Account
Logout
Search > Advanced form-based search
Search examples: ontological concepts - 'plant height' or 'regulation of gene expression'; gene names - 'GRP2' or 'TCP2'.
QTL ID: 'AQAA003' ; protein name: 'TBP1'
Ontology
GO:0005615
Search
Search:
Show 30 entries
Name
Description
URI
1
GO:0005615 (display)
extracellular space
That part of a multicellular organism outside the cells proper, usually taken to be outside the plasma membranes, and occupied by fluid.
http://purl.obolibrary.org/obo/GO_0005615 (in Sparql)
2
GO:0005615 (display)
extracellular space
http://purl.obolibrary.org/obo/GO_0005615 (in Sparql)
Showing 1 to 2 of 2 entries
Step1:
Select type of search between
Gene
Protein
QTL
PATHWAY
Ontology
Step2:
Enter the value
And search
Step3:
Display one result

Click to go up (more general in the ontology) and display results

Click to go down (more specific in the ontology) and display results

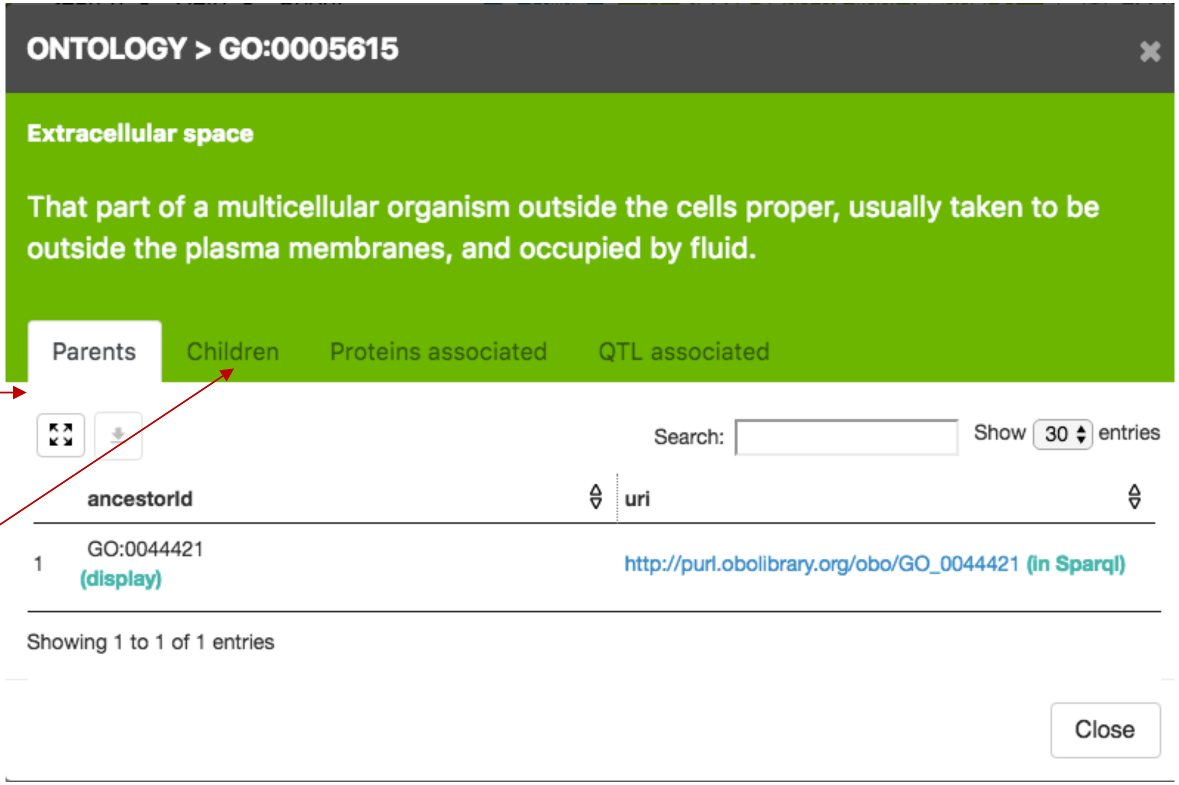

Display proteins having the search annotation GO:0005615

Select the protein to display (Q5Z710)

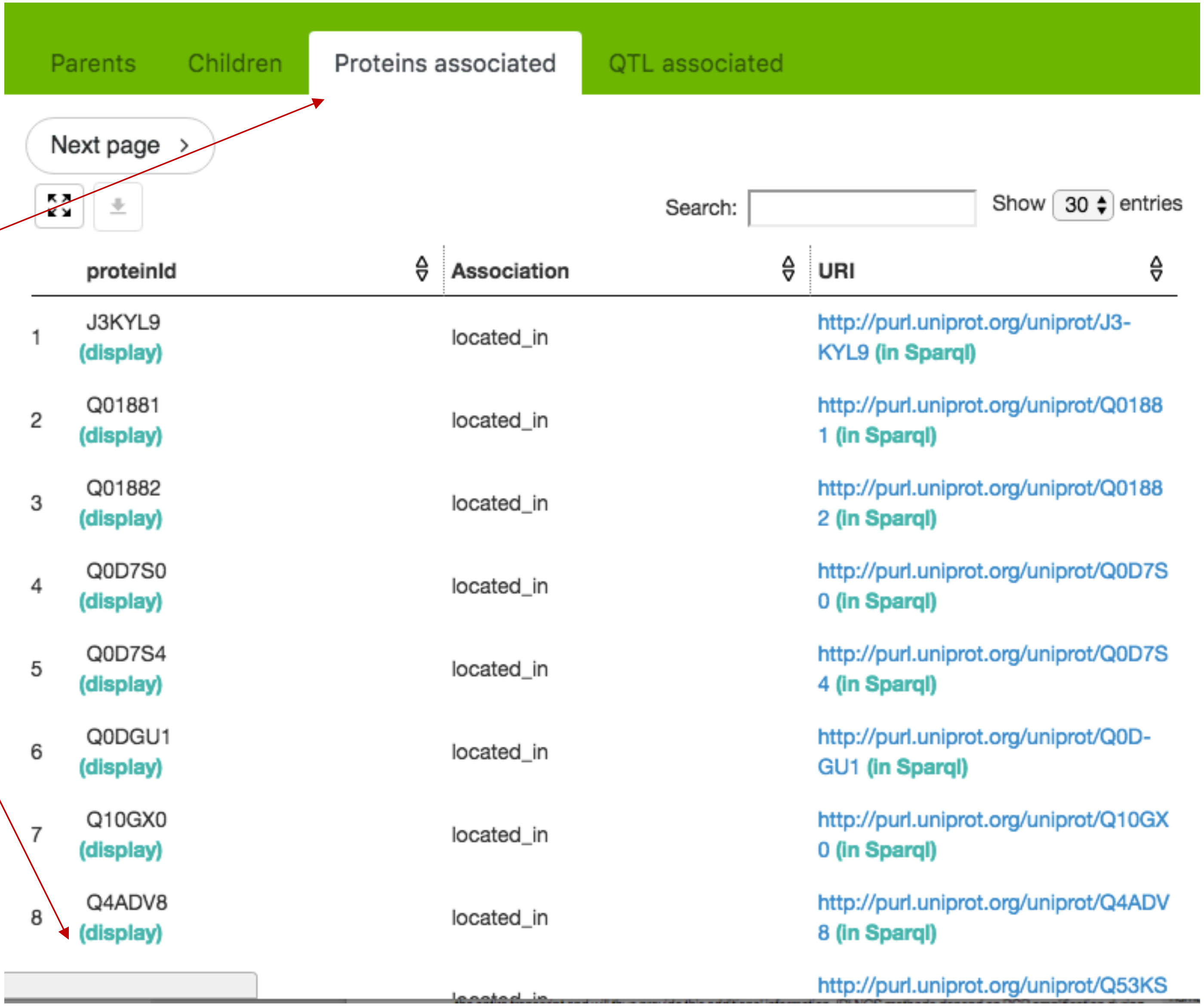

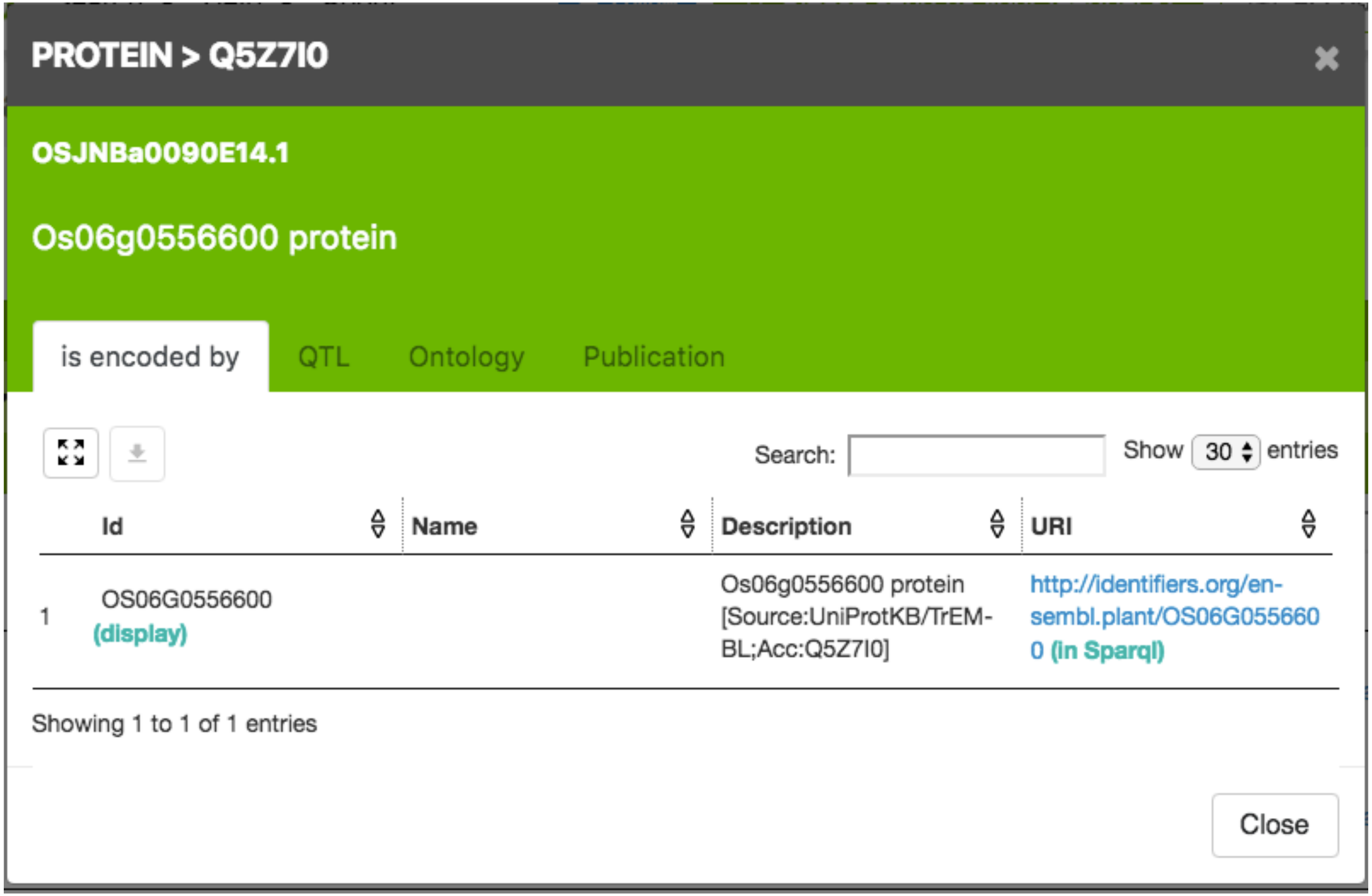
PROTEIN > Q5Z7I0
OSJNBa0090E14.1
Os06g0556600 protein
is encoded by
QTL
Ontology
Publication
Search:
Show 30 entries
Id
Name
Description
URI
1
OS06G0556600
(display)
Os06g0556600 protein [Source:UniProtKB/TrEMBL;Acc:Q5Z7I0]
http://identifiers.org/ensembl.plant/OS06G0556600 (in Sparql)
Showing 1 to 1 of 1 entries
Close

Quick Search

Search with keywords and browse AgroLD Knowledge Base

Use this tool

Advanced Search

Search with keywords, browse, and get answers to some biological questions

Use this tool

Explore Relationships

Search easily existing relationships between entities

Use this tool

SPARQL Query Editor

Edit and submit your SPARQL Queries to the sparql endpoint of AgroLD located

Use this tool

© AgroLD 2017

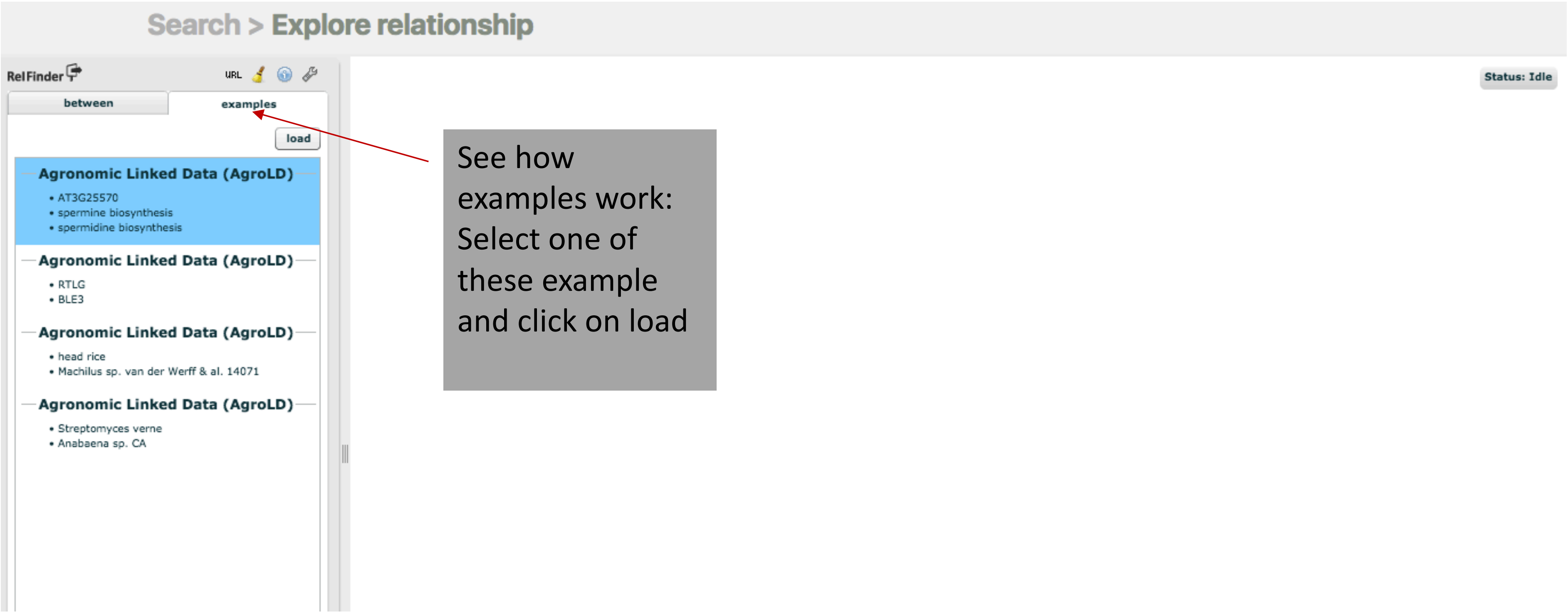
Search > Explore relationship
RelFinder
URL
between
examples
load
Agronomic Linked Data (AgroLD)
• AT3G25570
• spermine biosynthesis
• spermidine biosynthesis
Agronomic Linked Data (AgroLD)
• RTLG
• BLE3
Agronomic Linked Data (AgroLD)
• head rice
• Machilus sp. van der Werff & al. 14071
Agronomic Linked Data (AgroLD)
• Streptomyces verne
• Anabaena sp. CA
Status: Idle
See how examples work: Select one of these example and click on load

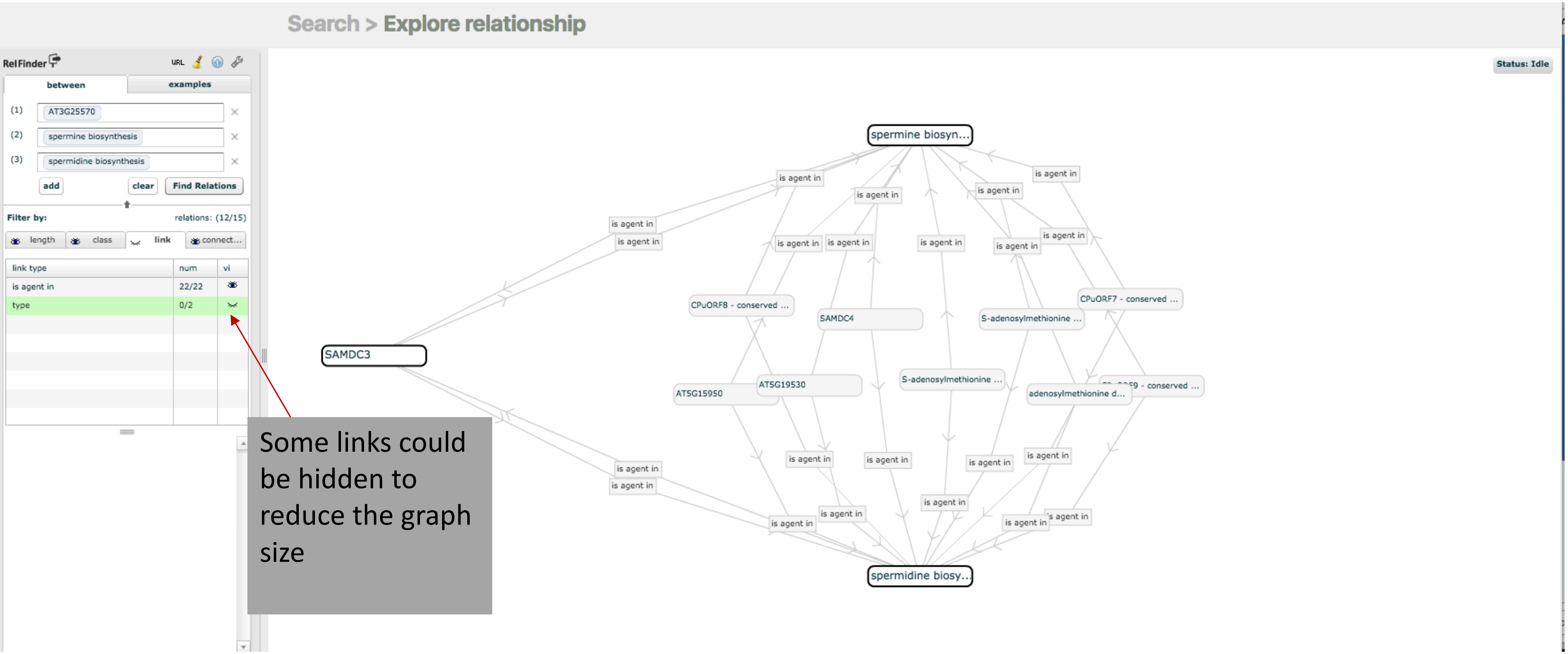
Search > Explore relationship
RelFinder
URL
between
examples
(1) AT3G25570
(2) spermine biosynthesis
(3) spermidine biosynthesis
add
clear
Find Relations
Filter by:
relations: (12/15)
length
class
link
connect...
link type
num
vi
is agent in
22/22
type
0/2
Status: Idle
spermine biosyn...
SAMDC3
spermidine biosy...
CPuORF8 - conserved ...
SAMDC4
S-adenosylmethionine ...
CPuORF7 - conserved ...
AT5G15950
AT5G19530
S-adenosylmethionine ...
adenosylmethionine d...
is agent in
Some links could be hidden to reduce the graph size

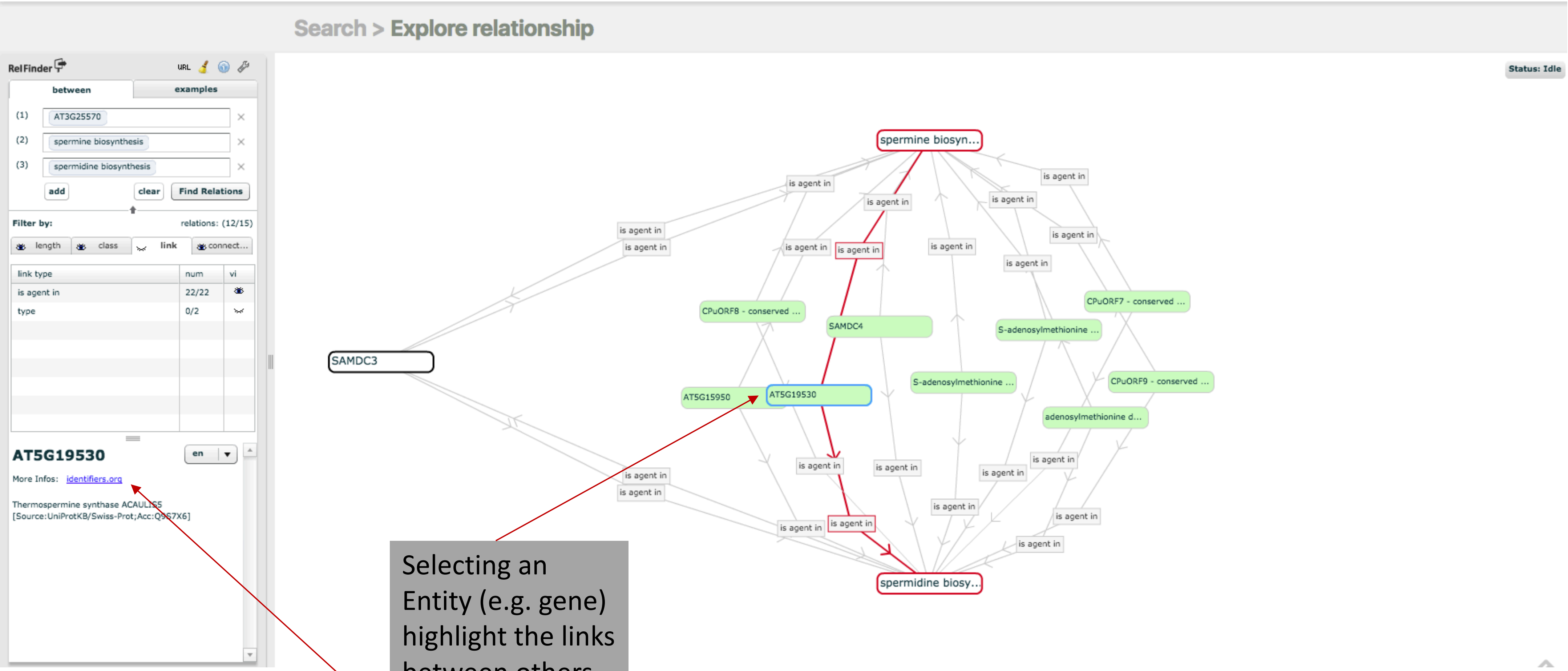

Selecting an Entity (e.g. gene) highlight the links between others entities.

More results are display by following this link

Explore the knowledge graphs with your own keywords

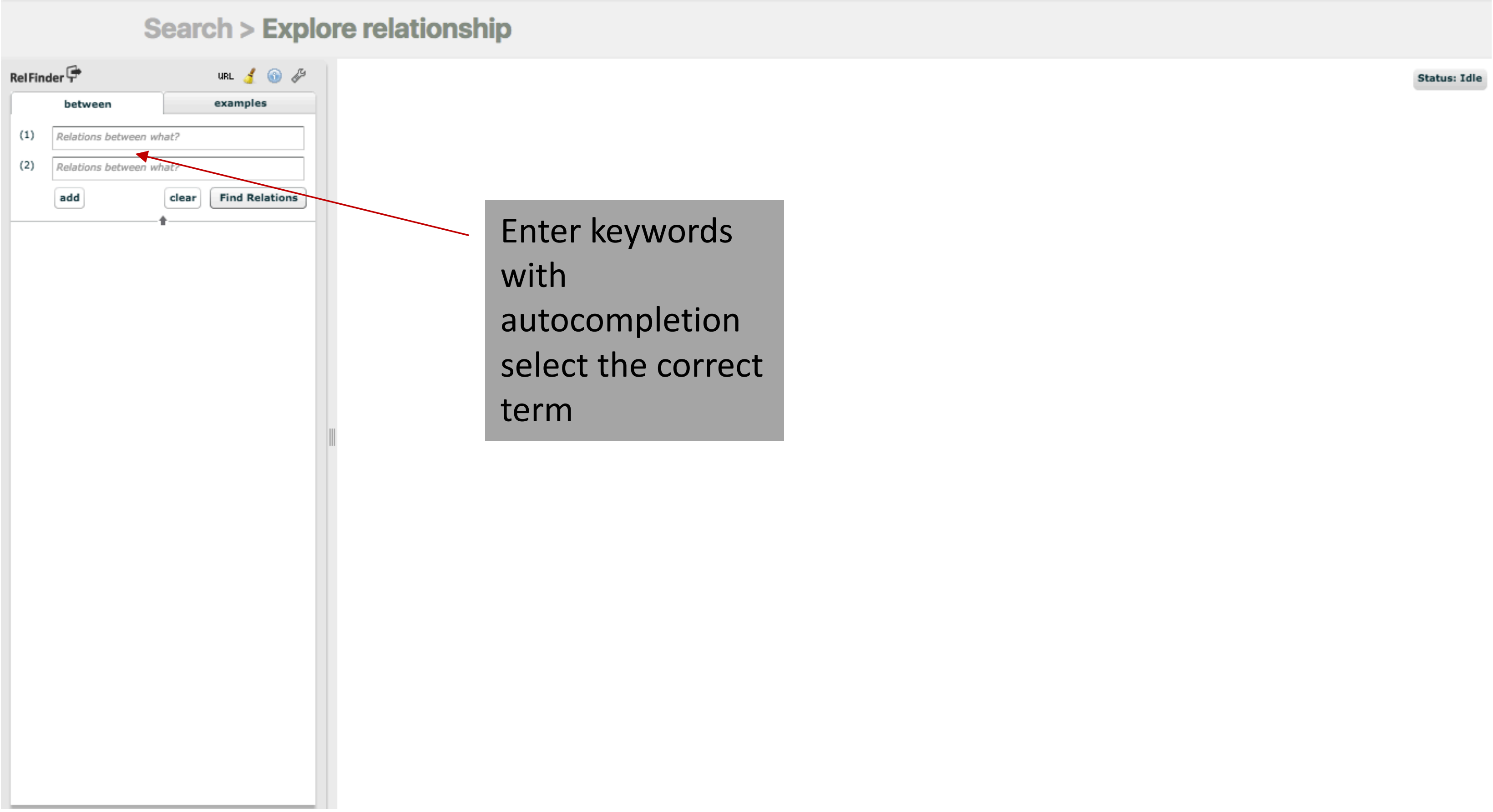

Enter keywords with autocompletion select the correct term

Quick Search

Search with keywords and browse AgroLD Knowledge Base

Use this tool

Advanced Search

Search with keywords, browse, and get answers to some biological questions

Use this tool

Explore Relationships

Search easily existing relationships between entities

Use this tool

SPARQL Query Editor

Edit and submit your SPARQL Queries to the sparql endpoint of AgroLD located

Use this tool

© AgroLD 2017

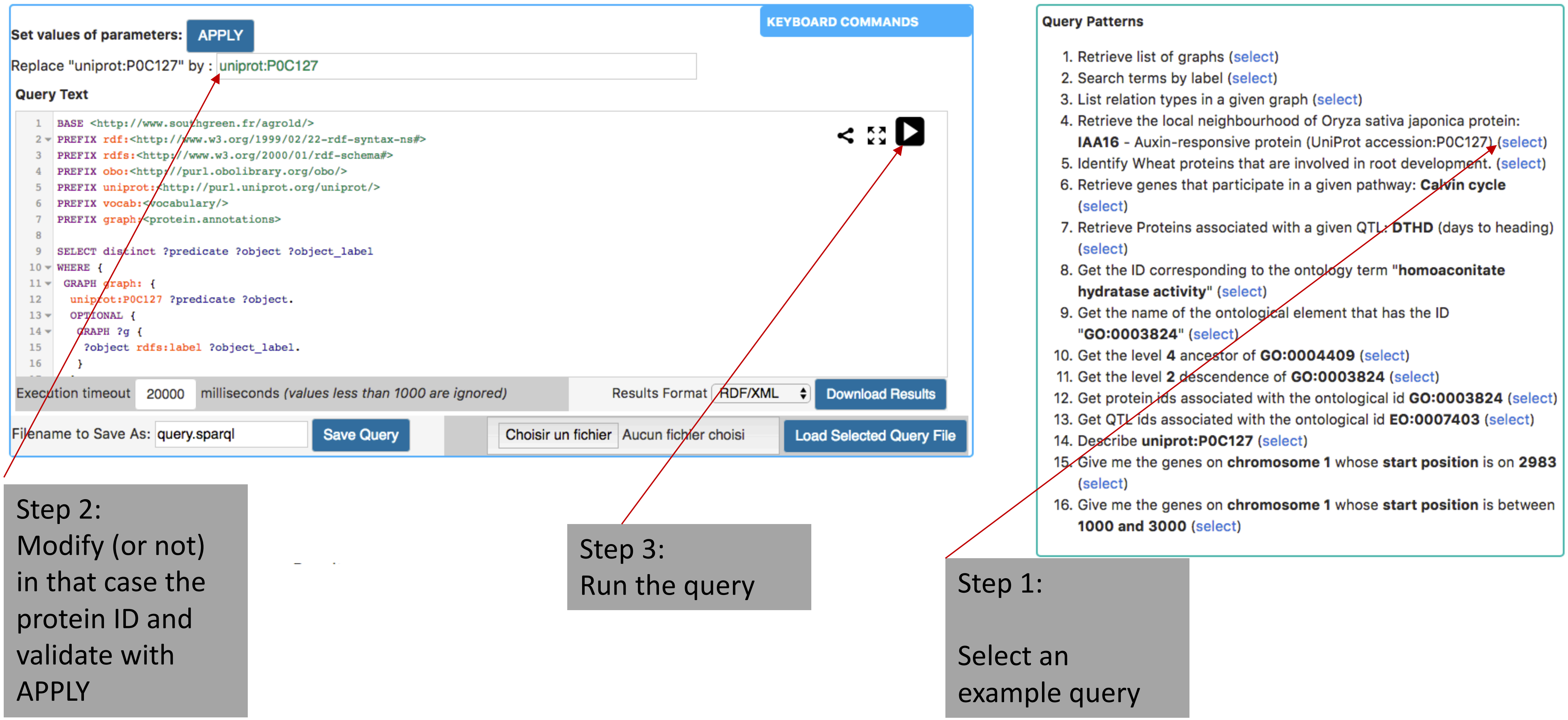
Set values of parameters: APPLY
KEYBOARD COMMANDS
Replace "uniprot:P0C127" by : uniprot:P0C127
Query Text
BASE <http://www.southgreen.fr/agrold/>
PREFIX rdf:<http://www.w3.org/1999/02/22-rdf-syntax-ns#>
PREFIX rdfs:<http://www.w3.org/2000/01/rdf-schema#>
PREFIX obo:<http://purl.obolibrary.org/obo/>
PREFIX uniprot:<http://purl.uniprot.org/uniprot/>
PREFIX vocab:<vocabulary/>
PREFIX graph:<protein.annotations>
SELECT distinct ?predicate ?object ?object_label
WHERE {
GRAPH graph: {
uniprot:P0C127 ?predicate ?object.
OPTIONAL {
GRAPH ?g {
?object rdfs:label ?object_label.
}
Execution timeout 20000 milliseconds (values less than 1000 are ignored)
Results Format RDF/XML
Download Results
Filename to Save As: query.sparql
Save Query
Choisir un fichier Aucun fichier choisi
Load Selected Query File
Query Patterns
1. Retrieve list of graphs (select)
2. Search terms by label (select)
3. List relation types in a given graph (select)
4. Retrieve the local neighbourhood of Oryza sativa japonica protein: IAA16 - Auxin-responsive protein (UniProt accession:P0C127) (select)
5. Identify Wheat proteins that are involved in root development. (select)
6. Retrieve genes that participate in a given pathway: Calvin cycle (select)
7. Retrieve Proteins associated with a given QTL: DTHD (days to heading) (select)
8. Get the ID corresponding to the ontology term "homoaconitate hydratase activity" (select)
9. Get the name of the ontological element that has the ID "GO:0003824" (select)
10. Get the level 4 ancestor of GO:0004409 (select)
11. Get the level 2 descendence of GO:0003824 (select)
12. Get protein ids associated with the ontological id GO:0003824 (select)
13. Get QTL ids associated with the ontological id EO:0007403 (select)
14. Describe uniprot:P0C127 (select)
15. Give me the genes on chromosome 1 whose start position is on 2983 (select)
16. Give me the genes on chromosome 1 whose start position is between 1000 and 3000 (select)
Step 2: Modify (or not) in that case the protein ID and validate with APPLY
Step 3: Run the query
Step 1: Select an example query

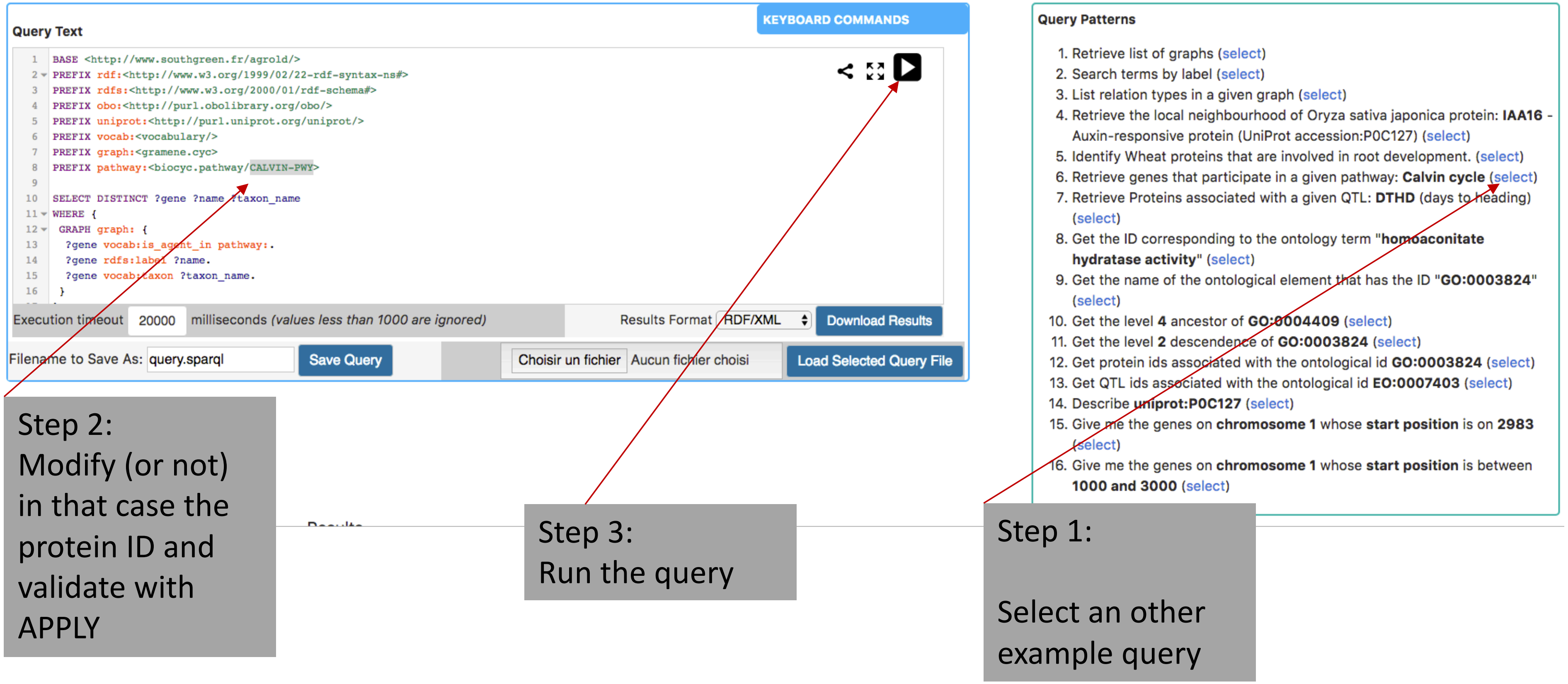
Query Text
KEYBOARD COMMANDS
BASE <http://www.southgreen.fr/agrold/>
PREFIX rdf:<http://www.w3.org/1999/02/22-rdf-syntax-ns#>
PREFIX rdfs:<http://www.w3.org/2000/01/rdf-schema#>
PREFIX obo:<http://purl.obolibrary.org/obo/>
PREFIX uniprot:<http://purl.uniprot.org/uniprot/>
PREFIX vocab:<vocabulary/>
PREFIX graph:<gramene.cyc>
PREFIX pathway:<biocyc.pathway/CALVIN-PWY>
SELECT DISTINCT ?gene ?name ?taxon_name
WHERE {
GRAPH graph: {
?gene vocab:is_agent_in pathway:.
?gene rdfs:label ?name.
?gene vocab:taxon ?taxon_name.
}
Execution timeout 20000 milliseconds (values less than 1000 are ignored)
Results Format RDF/XML
Download Results
Filename to Save As: query.sparql
Save Query
Choisir un fichier Aucun fichier choisi
Load Selected Query File
Query Patterns
1. Retrieve list of graphs (select)
2. Search terms by label (select)
3. List relation types in a given graph (select)
4. Retrieve the local neighbourhood of Oryza sativa japonica protein: IAA16 - Auxin-responsive protein (UniProt accession:P0C127) (select)
5. Identify Wheat proteins that are involved in root development. (select)
6. Retrieve genes that participate in a given pathway: Calvin cycle (select)
7. Retrieve Proteins associated with a given QTL: DTHD (days to heading) (select)
8. Get the ID corresponding to the ontology term "homoaconitate hydratase activity" (select)
9. Get the name of the ontological element that has the ID "GO:0003824" (select)
10. Get the level 4 ancestor of GO:0004409 (select)
11. Get the level 2 descendence of GO:0003824 (select)
12. Get protein ids associated with the ontological id GO:0003824 (select)
13. Get QTL ids associated with the ontological id EO:0007403 (select)
14. Describe uniprot:P0C127 (select)
15. Give me the genes on chromosome 1 whose start position is on 2983 (select)
16. Give me the genes on chromosome 1 whose start position is between 1000 and 3000 (select)
Step 2:
Modify (or not) in that case the protein ID and validate with APPLY
Step 3:
Run the query
Step 1:
Select an other example query

Results

Raw Response | Table | Pivot Table

Search: Show 50 entries

	gene	name	taxon_name
1	http://www.southgreen.fr/agrold/tigr.locus/LOC_Os01g02880.1	fructose-bisphosphate aldolase	obo:NCBITaxon_39947
2	http://www.southgreen.fr/agrold/tigr.locus/LOC_Os01g05490.1	triose-phosphate isomerase	obo:NCBITaxon_39947
3	http://www.southgreen.fr/agrold/tigr.locus/LOC_Os01g36090.1	ribose-5-phosphate isomerase	obo:NCBITaxon_39947
4	http://www.southgreen.fr/agrold/tigr.locus/LOC_Os01g39280.1	pyridoxin biosynthesis protein ER1, putative	obo:NCBITaxon_39947
5	http://www.southgreen.fr/agrold/tigr.locus/LOC_Os01g58020.1	ribulose-bisphosphate carboxylase	obo:NCBITaxon_39947
6	http://www.southgreen.fr/agrold/tigr.locus/LOC_Os01g58610.1	phosphoglycerate kinase	obo:NCBITaxon_39947
7	http://www.southgreen.fr/agrold/tigr.locus/LOC_Os01g62420.1	triosephosphate isomerase, cytosolic, putative, expressed	obo:NCBITaxon_39947
8	http://www.southgreen.fr/agrold/tigr.locus/LOC_Os01g64660.1	phosphoric ester hydrolase	obo:NCBITaxon_39947

Follow the link

More details

Follow the link to the original source

About: fructose-bisphosphate aldolase at Agrold.org

An Entity of Type: in URI : http://www.southgreen.fr/agrold/tigr.locus/LOC_Os01g02880.1

Property	Value
vocabulary:develops_from	<http://identifiers.org/ricegap/LOC_Os01g02880>
vocabulary:is_agent_in	<http://www.southgreen.fr/agrold/biocyc.pathway/CALVIN-PWY><br><http://www.southgreen.fr/agrold/biocyc.pathway/GLUCONEO-PWY><br><http://www.southgreen.fr/agrold/biocyc.pathway/GLYCOLYSIS><br><http://www.southgreen.fr/agrold/biocyc.pathway/P185-PWY><br><http://www.southgreen.fr/agrold/biocyc.pathway/PWY-1042><br><http://www.southgreen.fr/agrold/biocyc.pathway/PWY-3801>
rdfs:label	fructose-bisphosphate aldolase
vocabulary:taxon	<http://purl.obolibrary.org/obo/NCBITaxon_39947>
rdf:type	<http://www.southgreen.fr/agrold/resource/Gene>

List genes involve in this pathway

Thanks for your support !

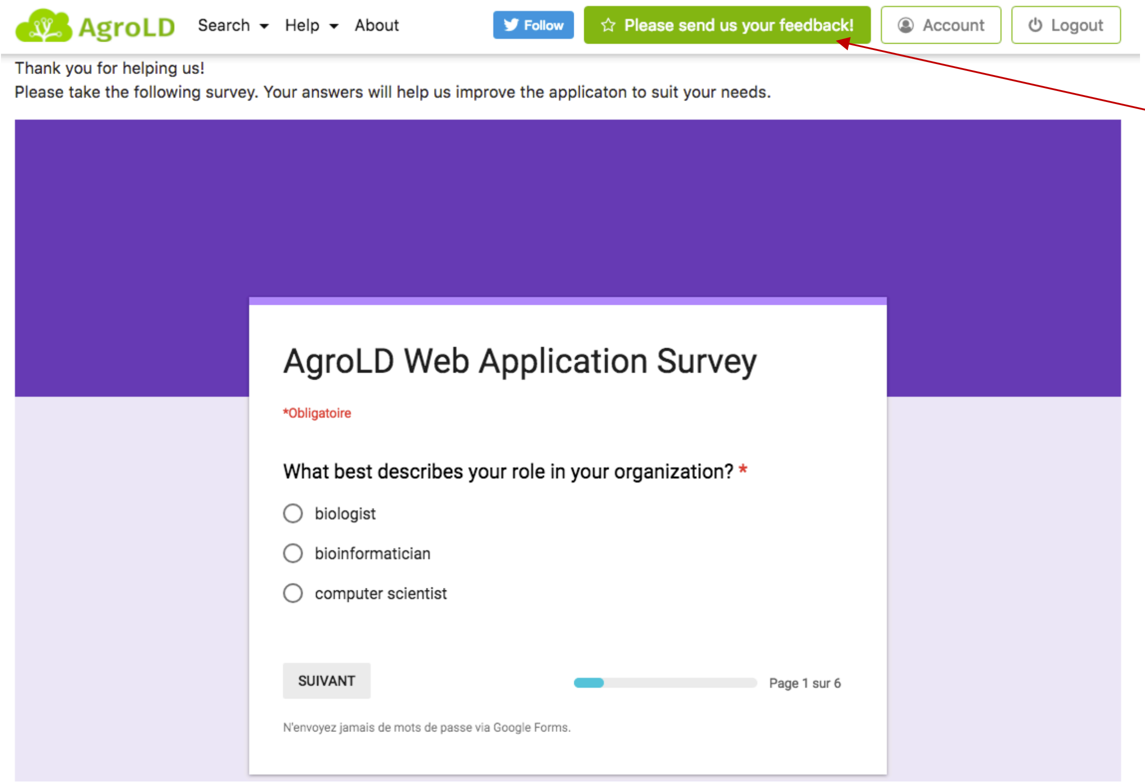

Please take 5 min to fill up our survey !

http://volvestre.cirad.fr:8080/agrold/survey.jsp
