# Supplementary material for: Agronomic Linked Data (AgroLD): A knowledge-based system to enable integrative biology in agronomy
Source: PLoS One. 2018 Nov 30;13(11):e0198270. doi: 10.1371/journal.pone.0198270 (PMC6269127; doi:10.1371/journal.pone.0198270)
Supplement: S2 File — Report of 3 sessions evaluating the AgroLD user interfaces. (PDF) [file pone.0198270.s002.pdf]

Results of the AgroLD online survey

A selection of results

What best describe your role in your organisation ?
20 answers

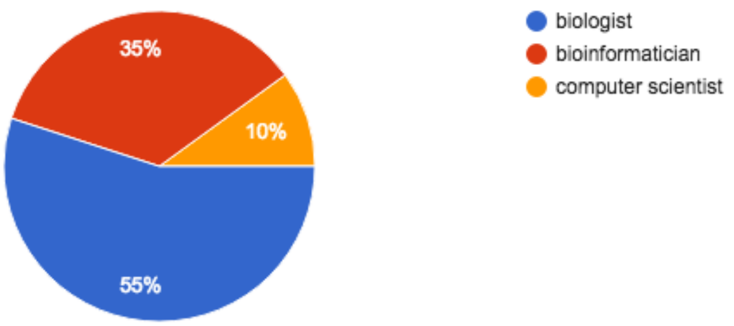

General questions about the platform

What features can be useful for you ?
20 answers

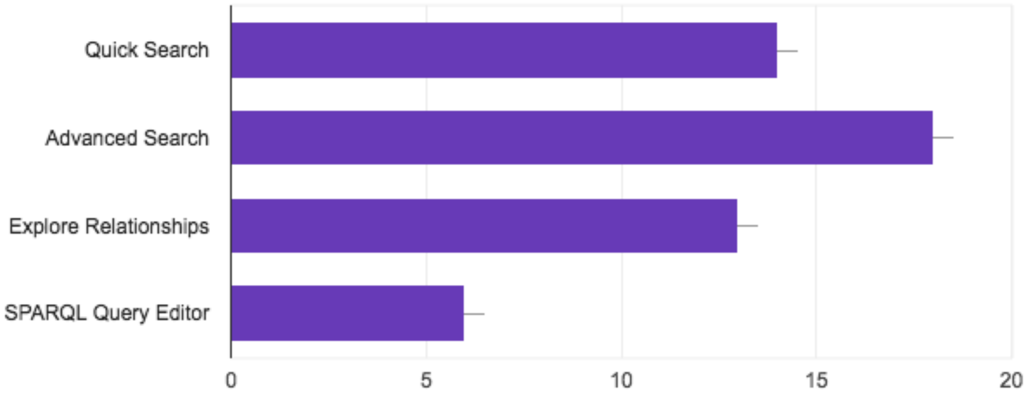

Do you think you could use the platform frequently ?

20 answers

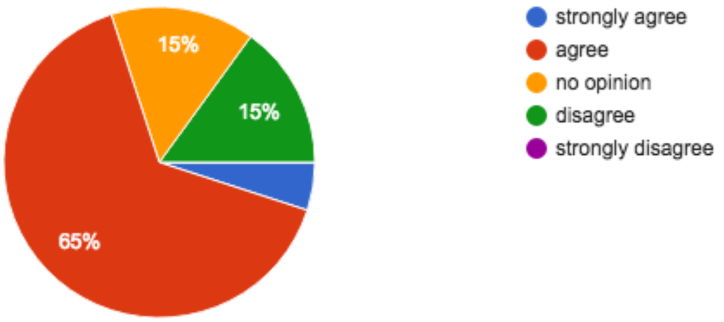

Do you think the platform is easy to use ?
20 answers

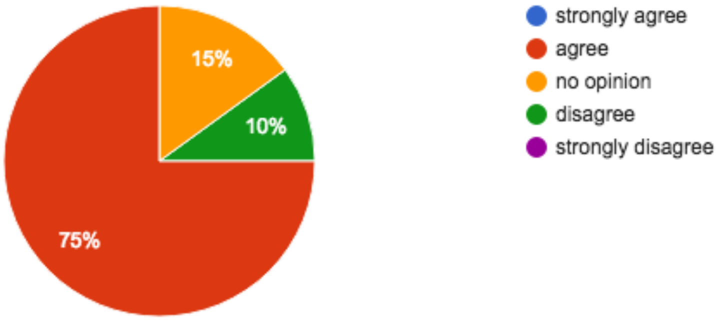

Do you think you would need some technical support for some features of the platform ?
20 answers

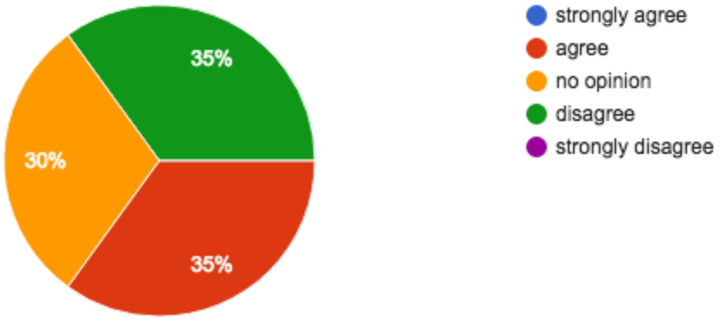

Do you found the various functions were well integrated ?
20 answers

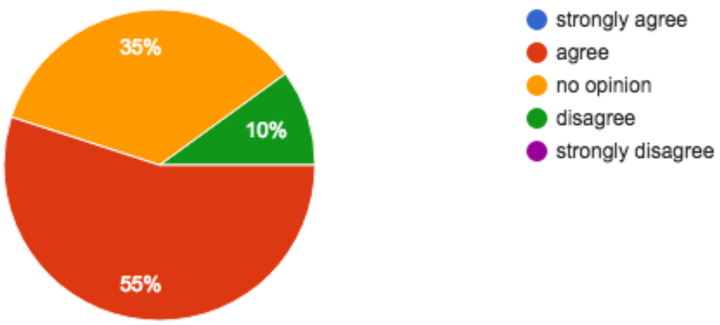

Questions about Quick Search

It is easy to enter and submit your query
20 answers

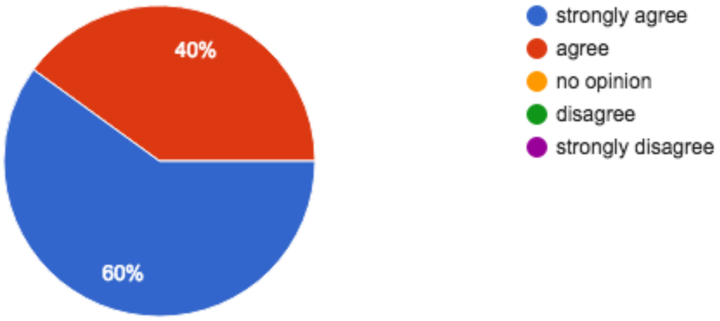

Do you think people will use this system ?
20 answers

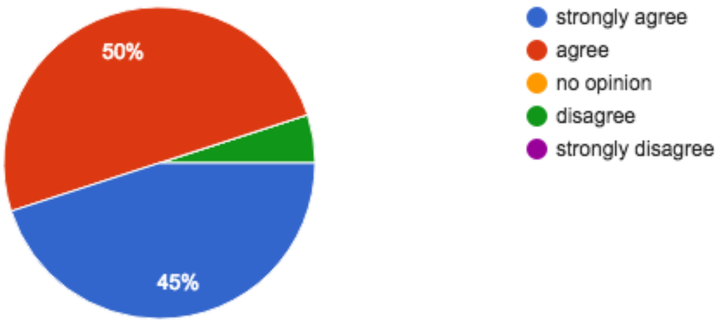

How much time did it take to find your answer ?
20 answers

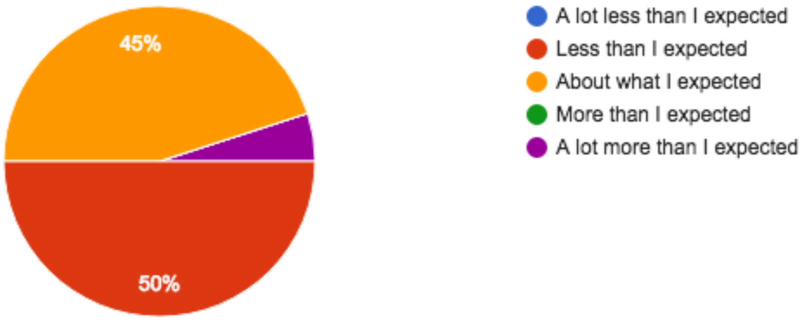

The results are well presented
20 answers

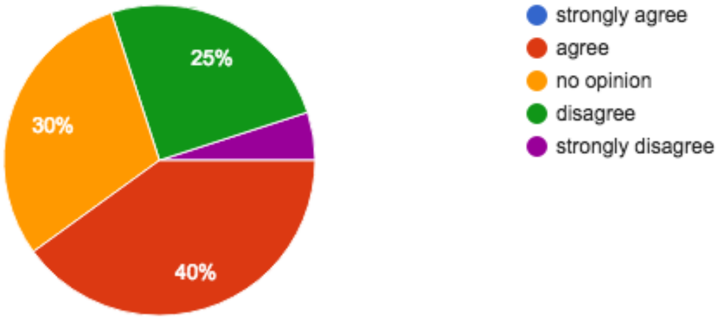

Do you have any suggestion or comment?

5 réponses

The results are not very intuitive for biologists

NO RESULTS !

Les résultats suite à une "quick search" peuvent être améliorés. il faudrait rajouter le nom de la source (en plus du lien). Serait il possible de savoir si on a trouvé un résultat (section gene, pathway, ontology...) comme pour la recherche avancée où l information est bien présentée.

column names are not user friendly and page is not the same website anymore: confusing

The output pages should be more readable for users

About Advanced Search

It is easy to enter and submit your query
20 answers

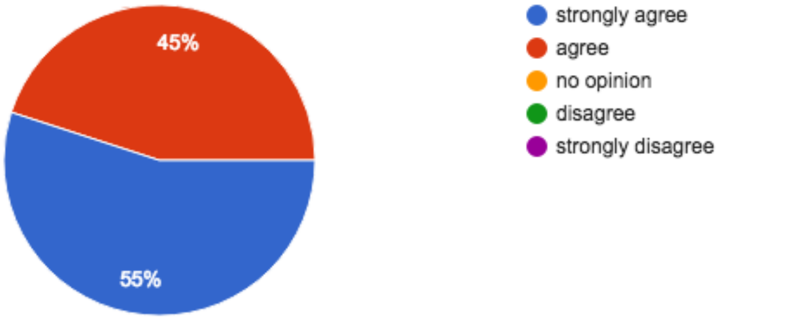

Do you think people would learn to use this system very quickly ?
20 answers

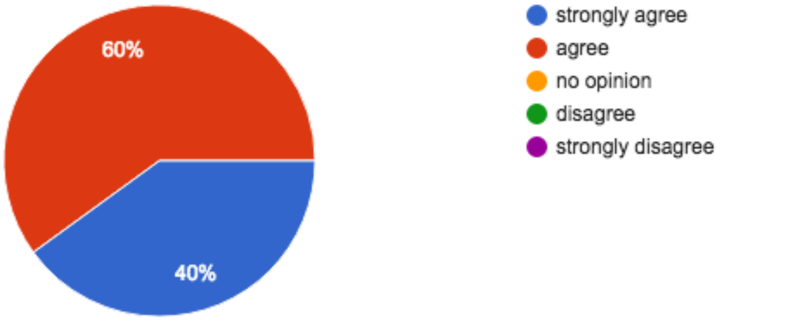

How much time did it take to find your answer ?
20 answers

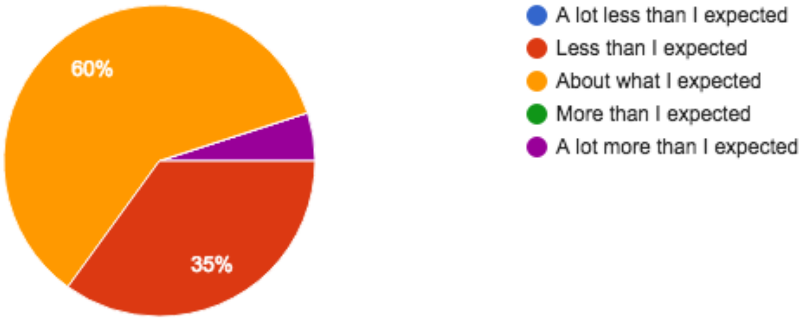

The results are well presented
20 answers

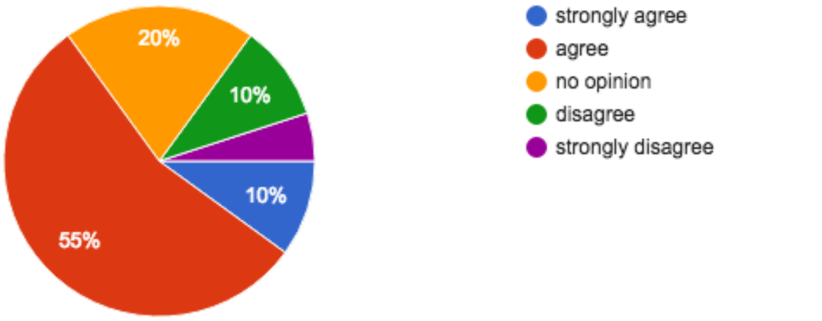

Did the results that you get are correct ?
20 answers

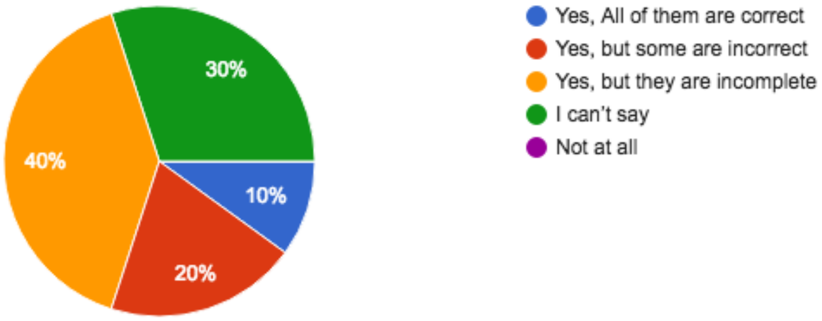

About Explore relationship

It is easy to enter and submit your query
20 answers

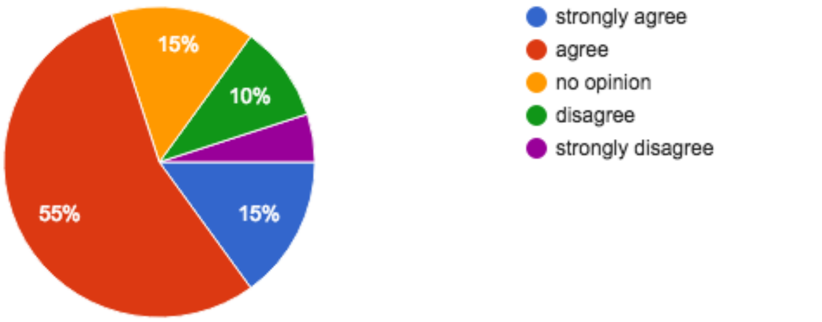

Do you think people would learn to use this system very quickly ?

20 answers

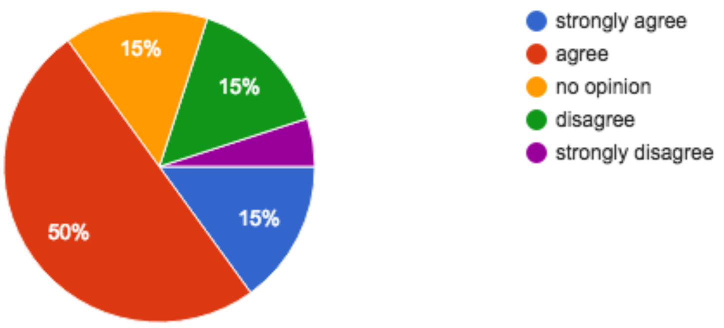

How much time did it take to find your answer ?
20 answers

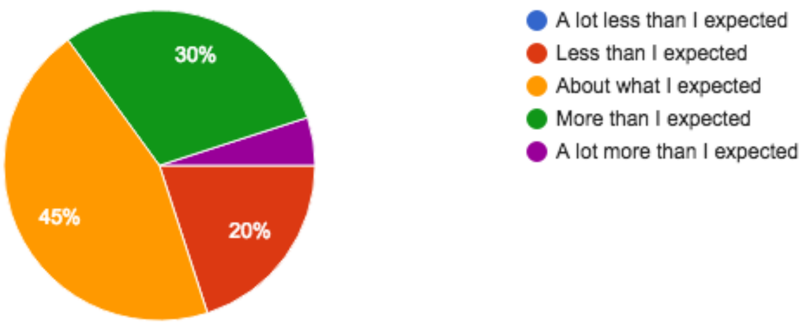

The results are well presented
20 answers

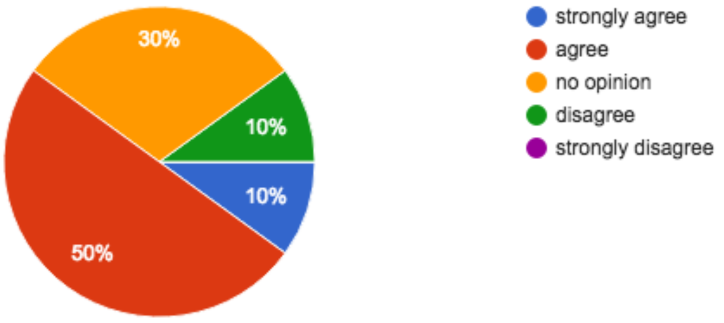

About Sparql Query

It is easy to enter and submit your query
20 answers

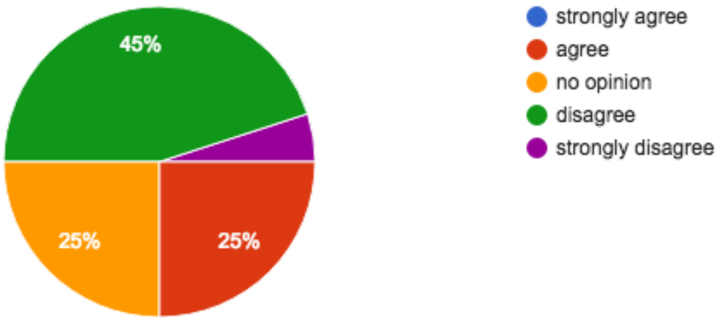

Do you think people would learn to use this system very quickly ?
20 answers

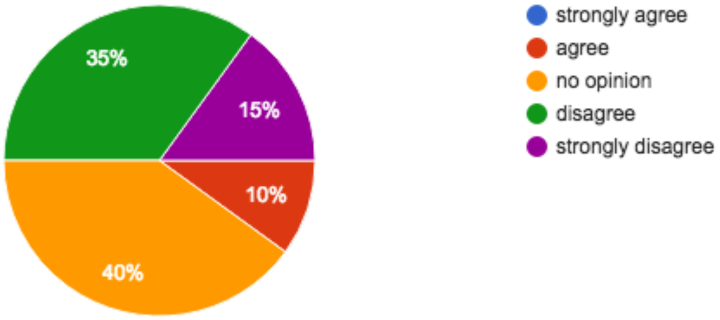

How much time did it take to find your answer ?
20 answers

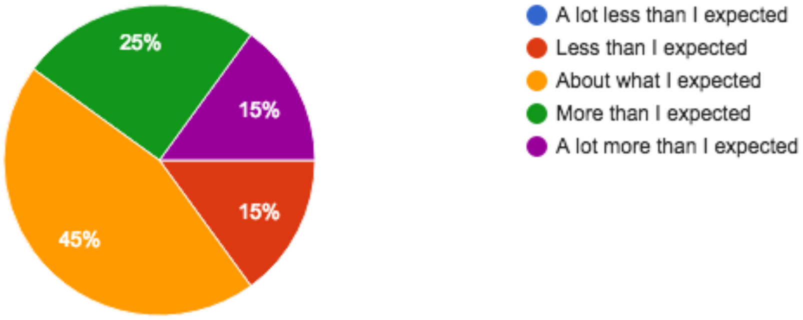

The results are well presented
20 answers

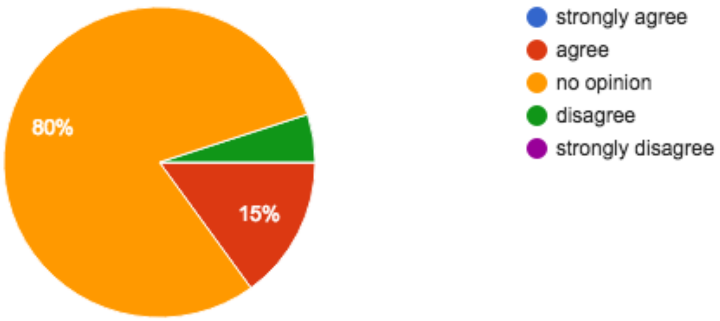
80%
15%
strongly agree
agree
no opinion
disagree
strongly disagree
