# Supplementary material for: Agronomic Linked Data (AgroLD): A knowledge-based system to enable integrative biology in agronomy
Source: PLoS One. 2018 Nov 30;13(11):e0198270. doi: 10.1371/journal.pone.0198270 (PMC6269127; doi:10.1371/journal.pone.0198270)
Supplement: S3 File — Example of SPARQL queries showing the benefits of property path algorithm, and complex queries. (PDF) [file pone.0198270.s003.pdf]

Identify Wheat proteins that are involved in root development.

S3_Q1 # Query without using poperty path

```
BASE <http://www.southgreen.fr/agrold/>
PREFIX rdf:<http://www.w3.org/1999/02/22-rdf-syntax-ns#>
PREFIX rdfs:<http://www.w3.org/2000/01/rdf-schema#>
PREFIX obo:<http://purl.obolibrary.org/obo/>
PREFIX taxon:<http://purl.obolibrary.org/obo/NCBITaxon_>
PREFIX uniprot:<http://purl.uniprot.org/uniprot/>
PREFIX vocab:<vocabulary/>
PREFIX graph:<protein.annotations>

SELECT distinct ?protein ?name ?evidence ?evidence_label ?evidence_code
WHERE {
 GRAPH graph: {
   {
    ?protein vocab:taxon taxon:4565.
    ?protein rdfs:label ?name.
     {
      ?protein ?p obo:GO_0048364.
      ?protein vocab:has_annotation ?bp.
      ?bp rdf:subject ?protein.
      ?bp rdf:object obo:GO_0048364.
      ?bp vocab:evidence_code ?evidence_code.
      ?bp vocab:evidence ?evidence.
     } UNION {
      ?protein ?p obo:GO_2000280.
      ?bp rdf:subject ?protein.
      ?protein vocab:has_annotation ?bp.
      ?bp rdf:object obo:GO_2000280.
      ?bp vocab:evidence_code ?evidence_code.
      ?bp vocab:evidence ?evidence.
     }
   }
 }
   GRAPH ?g {
   ?evidence rdfs:label ?evidence_label.

   }
}
```

RESULTS = 73 entries

S3_Q2 # Query using Property path

```
BASE <http://www.southgreen.fr/agrold/>
PREFIX rdf:<http://www.w3.org/1999/02/22-rdf-syntax-ns#>
PREFIX rdfs:<http://www.w3.org/2000/01/rdf-schema#>
PREFIX obo:<http://purl.obolibrary.org/obo/>
PREFIX taxon:<http://purl.obolibrary.org/obo/NCBITaxon_>
PREFIX uniprot:<http://purl.uniprot.org/uniprot/>
PREFIX vocab:<vocabulary/>
PREFIX graph2:<protein.annotations>
PREFIX graph1:<go>
SELECT distinct ?protein ?name ?label ?evidence ?evidence_label ?evidence_code
WHERE {
 GRAPH graph1: {
  {
   ?term rdfs:subClassOf* obo:GO_0048364.
   ?term rdfs:label ?label.
 } UNION {
   ?term rdfs:subClassOf* obo:GO_2000280.
  ?term rdfs:label ?label.
   }
  }
 GRAPH graph2: {
   ?protein vocab:taxon taxon:4565.
   ?protein rdfs:label ?name.
   ?protein ?p ?term.
   ?protein vocab:has_annotation ?bp.
   ?bp rdf:subject ?protein.
   ?bp rdf:object ?term.
   ?bp vocab:evidence_code ?evidence_code.
   ?bp vocab:evidence ?evidence.
  }
 GRAPH ?g {
  ?evidence rdfs:label ?evidence_label.

  }
}
```

RESULT= 137 entries

S3_Q3 ### Retrieve Individuals which have SNP variant effect "NON_SYNONYMOUS_CODING" identify into proteins associated with a QTL

```
BASE <http://www.southgreen.fr/agrold/>
PREFIX rdf:<http://www.w3.org/1999/02/22-rdf-syntax-ns#>
PREFIX rdfs:<http://www.w3.org/2000/01/rdf-schema#>
```

```
PREFIX obo:<http://purl.obolibrary.org/obo/>
PREFIX uniprot:<http://purl.uniprot.org/uniprot/>
PREFIX vocab:<vocabulary/>
PREFIX graph1:<protein.annotations>
PREFIX graph2:<qtl.annotations>
PREFIX graph3:<sniplaydb>


SELECT distinct ?indiv ?id ?name ?msu ?snpeff
WHERE {
GRAPH graph1: {
  ?id vocab:has_trait ?to.
  ?id rdfs:label ?name.
?id vocab:has_synonym ?syn.
FILTER(REGEX(?syn,'^.*LOC.Os.*' ))
      BIND (IF(isURI(?syn),
    ?syn,
        URI(CONCAT("http://www.identifiers.org/ricegap/",
            ENCODE_FOR_URI(?syn)))
    ) AS ?msu )

 }
  GRAPH graph2: {
  qtl: vocab:has_trait ?to.
 }
GRAPH graph3:{
  ?msu vocab:has_effect ?snpeff.
    FILTER(REGEX(?snpeff,'^NON_SYNONYMOUS_CODING' ))
   ?msu vocab:part_of ?indiv.
    FILTER(REGEX(?indiv,'^http://www.southgreen.fr/agrold/sniplay.individual/')).
}
}

ORDER BY ?indiv
```
