# Supplementary material for: Agronomic Linked Data (AgroLD): A knowledge-based system to enable integrative biology in agronomy
Source: PLoS One. 2018 Nov 30;13(11):e0198270. doi: 10.1371/journal.pone.0198270 (PMC6269127; doi:10.1371/journal.pone.0198270)
Supplement: S1 Table — (PDF) [file pone.0198270.s004.pdf]

Dataset	graph name	Triples	Classes	Entities	Properties
orygenesdb.o.s.japonica	http://www.southgreen.fr/agrold/orygenesdb.o.s.japonica	6 170 076	17	979 439	19
orygenesdb.o.s.indica	http://www.southgreen.fr/agrold/orygenesdb.o.s.indica	3 925 042	10	602 860	10
orygenesdb.a.thaliana	http://www.southgreen.fr/agrold/orygenesdb.a.thaliana	4 673 224	9	686 940	11
greenphyldb	http://www.southgreen.fr/agrold/greenphyldb	662 346	2	52 411	22
tropgenedb	http://www.southgreen.fr/agrold/tropgenedb	20 073	5	4 514	11
sniplaydb	http://www.southgreen.fr/agrold/sniplaydb	16 204 042	1	2 317 311	14
qtaro.gene	http://www.southgreen.fr/agrold/qtaro.gene	14 639	1	1 008	10
oryzabase.gene	http://www.southgreen.fr/agrold/oryzabase.gene	164 126	1	13 086	19
protein.annotations	http://www.southgreen.fr/agrold/protein.annotations	5 649 921	2	420 312	21
qtl.annotations	http://www.southgreen.fr/agrold/qtl.annotations	570 829	2	59 636	18
gramene.qtl	http://www.southgreen.fr/agrold/gramene.qtl	61 429	1	8 216	8
gramene.gene	http://www.southgreen.fr/agrold/gramene.gene	4 466 067	1	409 765	15
gramene.cyc	http://www.southgreen.fr/agrold/gramene.cyc	98 512	2	16 424	5
OryzaTagLine	http://www.southgreen.fr/agrold/otl	282 217	1	32 022	16
uniprot.plants	http://www.southgreen.fr/agrold/uniprot.plants	50 442 318	1	3 311 251	10
**Total**		93 404 861	56	5 571 922	209
